# Direct synthesis of acyl fluorides from carboxylic acids using benzothiazolium reagents

**DOI:** 10.3762/bjoc.20.82

**Published:** 2024-04-23

**Authors:** Lilian M Maas, Alex Haswell, Rory Hughes, Matthew N Hopkinson

**Affiliations:** 1 Institute of Chemistry and Biochemistry, Freie Universität Berlin, Fabeckstrasse 34–36, 14195 Berlin, Germany; 2 School of Natural and Environmental Sciences, Newcastle University, Bedson Building, Newcastle upon Tyne, NE1 7RU, United Kingdom

**Keywords:** acyl fluorides, amides, benzothiazolium salts, carboxylic acids, deoxygenative reactions

## Abstract

2-(Trifluoromethylthio)benzothiazolium triflate (BT-SCF_3_) was used as deoxyfluorinating reagent for the synthesis of versatile acyl fluorides directly from the corresponding carboxylic acids. These acyl fluorides were reacted with amines in a one-pot protocol to form different amides, including dipeptides, under mild and operationally simple conditions in high yields. Mechanistic studies suggest that BT-SCF_3_ can generate acyl fluorides from carboxylic acids via two distinct pathways, which allows the deoxyfluorinating reagent to be employed in sub-stoichiometric amounts.

## Introduction

Acyl fluorides are attracting much attention as versatile reagents for different applications in organic synthesis. In addition to their use as sources of fluoride ions, they are most commonly employed as acylation reagents [1–3]. The strong C–F bond makes acyl fluorides relatively stable towards hydrolysis and easier to handle than other acyl halides [4–8]. Their reactions with nucleophiles are typically less violent than for the corresponding acyl chlorides with acyl fluorides exhibiting comparable electrophilicity to activated esters; however, with considerably fewer steric restrictions [9–10]. Acylations with acyl fluorides also typically proceed with fewer side-reactions while derivatives bearing an α-stereocentre generally undergo little racemisation [11–12]. The combination of all these properties mean that acyl fluorides can provide significant advantages over acyl chlorides, especially for challenging acylation reactions [13–14].

Nevertheless, acyl chlorides still dominate in the literature; however, the recent development of safer and more practical synthetic routes to acyl fluorides are inspiring greater interest in these compounds. Various synthetic approaches have been investigated with two main strategies being pursued: fluorine-transfer to acyl radicals and nucleophilic fluorination of acyl electrophiles [15]. The latter approach is the most intensively studied due to the easy accessibility of fluoride ions with many methods directly employing the parent carboxylic acid as substrate. These processes avoid an additional pre-functionalisation step and have been reported using a range of deoxyfluorinating reagents including (diethylamino)sulfur trifluoride (DAST) [16–18], bis(2-methoxyethyl)aminosulfur trifluoride (Deoxo-Fluor^®^) [10,19–20], (diethylamino)difluorosulfonium tetrafluoroborate (XtalFluor-E^®^) [21–24], (Me_4_N)SCF_3_ [9,25], pentafluoropyridine (PFP) [26] and cyanuric fluoride [27–28] among others [15].

**Figure 1:**
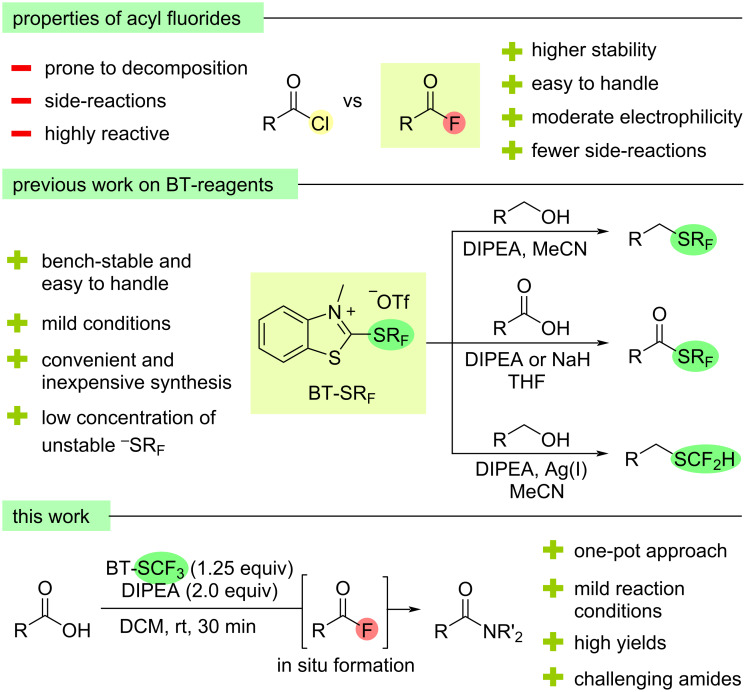
Advantages of acyl fluorides compared to acyl chlorides, previous work on BT-SR_F_ reagents [29–33] and a summary of this work on the BT-SCF_3_-mediated in situ formation of acyl fluorides and their use for the synthesis of amides.

Since 2019, our group has developed a series of 2-(fluoroalkylthio)benzothiazolium (BT-SR_F_) reagents for the deoxygenative transfer of SR_F_ (R_F_ = poly- or perfluoroalkyl) groups into organic molecules (Figure 1). In an initial report, the trifluoromethylthio-containing salt, BT-SCF_3_, was reacted with unactivated aliphatic alcohols to afford (trifluoromethyl)thioethers, while subsequent work focused on the direct deoxygenative synthesis of fluorinated thioesters from carboxylic acids [29–31]. In each case, the reactions proceeded smoothly under operationally simple conditions while BT-SCF_3_ and related BT-SR_F_ reagents are easy-to-handle solids that can be readily produced on a multigram scale from relatively inexpensive starting materials. During the optimisation studies for the latter process with carboxylic acid substrates, in addition to the desired (trifluoromethyl)thioester products, small amounts of the corresponding acyl fluorides were also observed as by-products. Given the increasing interest in acyl fluorides in organic synthesis and the attractive features of BT-SR_F_ salts as reagents for organofluorine chemistry, we considered whether optimisation of the reaction conditions could allow for the selective synthesis of acyl fluoride products directly from carboxylic acids. Here, we report the results of this study, which led to the development of a practical and high yielding methodology for the synthesis of acyl fluorides and their subsequent one-pot conversion into amides. Moreover, by virtue of BT-SCF_3_’s ability to deliver acyl fluorides via two distinct deoxyfluorination pathways, an efficient process could be achieved using only sub-stoichiometric amounts of the fluorinating reagent.

## Results and Discussion

In an initial test reaction, 4-methylbenzoic acid (**1a**) was reacted with 1.25 equiv of BT-SCF_3_ and 2.0 equiv of NaH in DCM under conditions similar to our previous reports on the deoxygenative trifluoromethylthiolation of carboxylic acids [31]. ^19^F NMR analysis of the crude reaction mixture after 2 h at rt revealed no conversion towards the desired acyl fluoride product **2a**, however, 30% of thioester **3a** was formed (internal standard: PhCF_3_, Table 1, entry 1). Pleasingly, changing the base to K_2_CO_3_ led to the formation of **2a** in 7% ^19^F NMR yield (Table 1, entry 2), while the selectivity of the reaction could be switched significantly upon employing organic amine bases (Table 1, entries 3 and 4). Using 2.0 equiv of diisopropylethylamine (DIPEA), **2a** could be obtained in quantitative ^19^F NMR yield although a reduction to 1.5 equiv led to a significant drop in efficiency, delivering the acyl fluoride in only 30% ^19^F NMR yield together with 45% of thioester **3a** (Table 1, entries 4 and 5). At this stage, we were interested in the reactivity of other BT-SR_F_ reagents developed in our group and tested three longer-chain derivatives under deoxyfluorination conditions. Employing BT-SC_4_F_9_ and BT-SC_8_F_17_, ^19^F NMR yields of **2a** of 84% and 81% were achieved (Table 1, entries 6 and 7), however, BT-SCF(CF_3_)_2_, which features a branched perfluoroalkyl chain, gave a comparatively moderate ^19^F NMR yield of 67% (Table 1, entry 8). BT-SCF_3_ still led to the highest ^19^F NMR yield of **2a** among all the tested reagents and was therefore used throughout the subsequent optimisation and scope studies. An interesting observation was made upon varying the equivalents of BT-SCF_3_. Rather than reducing the yield to 50% or lower, conducting the reaction with 0.5 equiv of BT-SCF_3_ provided **2a** in 55% ^19^F NMR yield, suggesting that each equivalent of the benzothiazolium reagent can deliver more than one equivalent of the acyl fluoride product (Table 1, entry 9). Although representing a considerable drop in efficiency compared to using 1.25 equiv of BT-SCF_3_, this observation provides an interesting insight into the reaction mechanism (vide infra). Changing the solvent from DCM to THF or MeCN resulted in no significant change in the efficiency of the reaction, whereas a ^19^F NMR yield of only 11% was achieved in DMF (Table 1, entries 10–12). Increasing the reaction concentration to 0.2 M in DCM led to a reduction in the ^19^F NMR yield of **2a** to 74% (Table 1, entry 13). Finally, optimisation of the reaction time revealed the starting material was completely converted after only 30 min at rt (Table 1, entry 14).

**Table 1:** Optimisation of the reaction conditions for the deoxygenative fluorination of 4-methylbenzoic acid using benzothiazolium reagents.

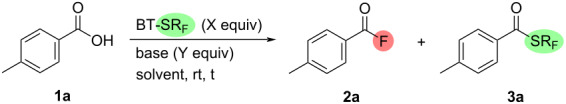						

Entry	R_F_ (X equiv)	Base (Y equiv)	Solvent (conc.)	*t* (h)	Yield **2a**^a^	Yield **3a**^a^

1	CF_3_ (1.25)	NaH (2.0)	DCM (0.1 M)	2	–	30
2	CF_3_ (1.25)	K_2_CO_3_ (2.0)	DCM (0.1 M)	2	7	37
3	CF_3_ (1.25)	NEt_3_ (2.0)	DCM (0.1 M)	2	96	traces
4	CF_3_ (1.25)	DIPEA (2.0)	DCM (0.1 M)	2	quant.	–
5	CF_3_ (1.25)	DIPEA (1.5)	DCM (0.1 M)	2	30	45
6	C_4_F_9_ (1.25)	DIPEA (2.0)	DCM (0.1 M)	2	84	–
7	C_8_F_17_ (1.25)	DIPEA (2.0)	DCM (0.1 M)	2	81	–
8	CF(CF_3_)_2_ (1.25)	DIPEA (2.0)	DCM (0.1 M)	2	67	–
9	CF_3_ (0.5)	DIPEA (2.0)	DCM (0.1 M)	2	55	–
10	CF_3_ (1.25)	DIPEA (2.0)	DMF (0.1 M)	2	11	–
11	CF_3_ (1.25)	DIPEA (2.0)	MeCN (0.1 M)	2	88	–
12	CF_3_ (1.25)	DIPEA (2.0)	THF (0.1 M)	2	91	–
13	CF_3_ (1.25)	DIPEA (2.0)	DCM (0.2 M)	2	74	–
14	CF_3_ (1.25)	DIPEA (2.0)	DCM (0.1 M)	0.5	quant.	–

^a^As internal standard for ^19^F NMR yields α,α,α-trifluorotoluene was used.

**Scheme 1:**
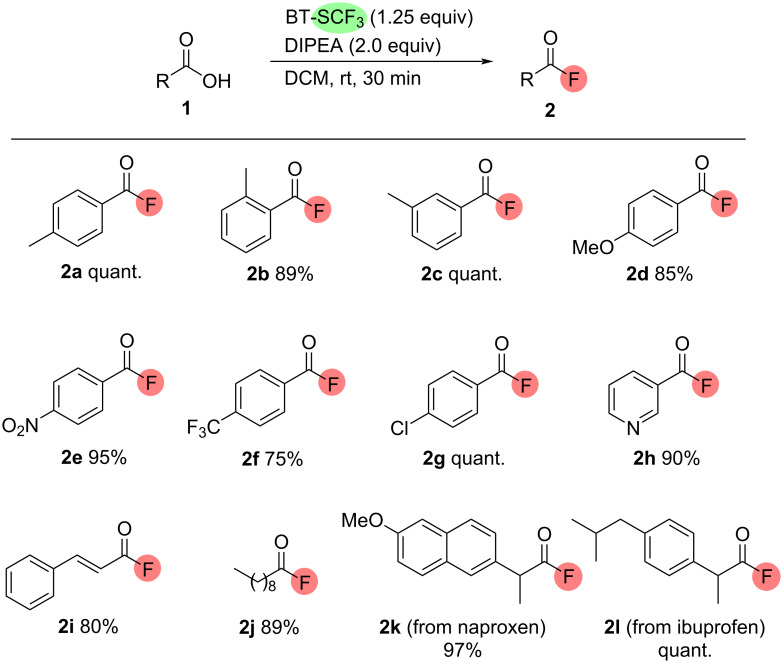
Scope of the BT-SCF_3_-mediated deoxygenative fluorination of carboxylic acids **1**. Reactions were performed on a 0.2 mmol scale. ^19^F NMR yields using α,α,α-trifluorotoluene as the internal standard.

With the optimised conditions in hand, the scope of the reaction was investigated to assess the practical utility of BT-SCF_3_-mediated deoxyfluorination as a method for preparing diverse acyl fluorides. As shown in Scheme 1, the reaction showed excellent functional group tolerance with a range of aromatic carboxylic acids **1**, delivering the corresponding acyl fluorides **2** in very good ^19^F NMR yields above 75% for all substrates tested. Both electron-withdrawing and electron-donating substituents were tolerated while substituents could be present at the *ortho-*, *meta-* or *para*-positions. The heteroaromatic acyl fluoride **2h** could be prepared efficiently while deoxyfluorination of representative olefinic and aliphatic carboxylic acids proceeded smoothly, affording cinnamoyl and decanoyl acyl fluorides **2i** and **2j** in 80% and 89% ^19^F NMR yields, respectively. Furthermore, the widely available drug molecules naproxen and ibuprofen could be efficiently converted into their acyl fluoride derivatives **2k** and **2l** in 97% and quantitative yields, respectively.

**Scheme 2:**
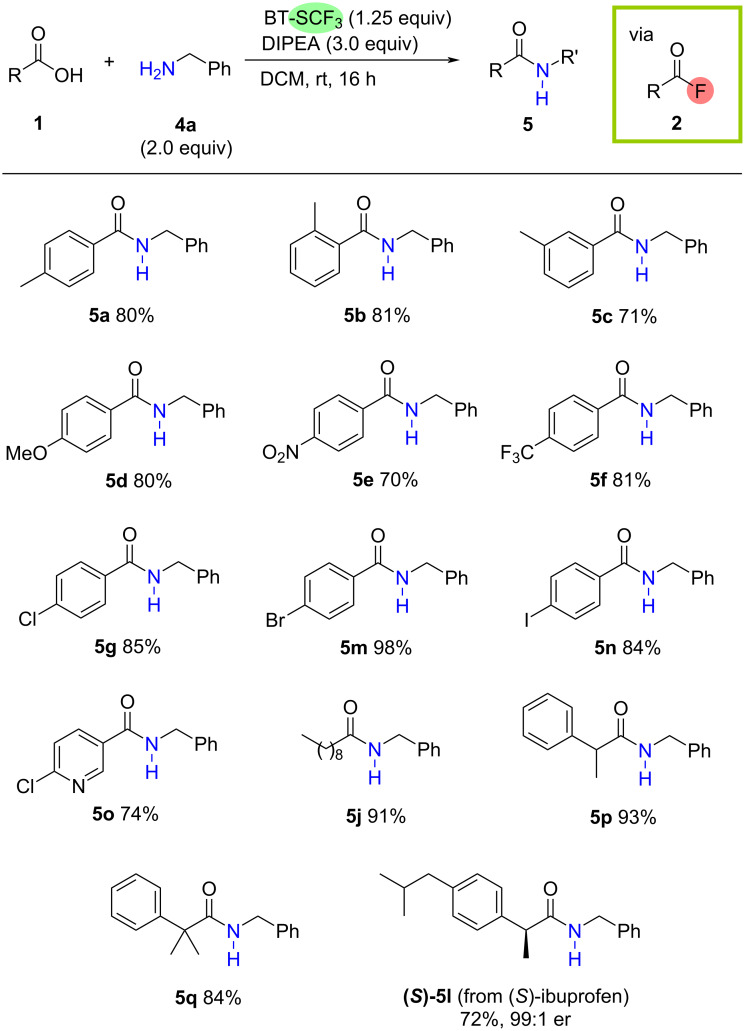
Scope of the one-pot BT-SCF_3_-mediated deoxygenative coupling of carboxylic acids and amines via acyl fluoride derivatives. Reactions conducted on a 0.5 mmol scale, isolated yields after column chromatography.

To improve the practicality of the methodology and to avoid the often unreliable isolation of acyl fluoride intermediates, we next considered whether BT-SCF_3_-mediated deoxyfluorination of carboxylic acids could be coupled with a subsequent acylation in an overall one-pot process. Selecting amines as nucleophilic coupling partners, a short optimisation study was carried out to identify suitable conditions compatible with the deoxyfluorination process. Pleasingly, adding 2.0 equiv of benzylamine (**4a**) to the standard reaction between 4-methylbenzoic acid (**1a**) and BT-SCF_3_ (1.25 equiv) in DCM (0.1 M) and increasing the amount of DIPEA to 3.0 equiv allowed for the efficient formation of the desired amide **5a** after 16 h at rt, which could be isolated in 80% yield after column chromatography. A survey of carboxylic acids **1** revealed that the one-pot approach is efficient for a variety of substitution profiles (Scheme 2). Aromatic acids bearing methyl substituents at the *para*-, *ortho*- or *meta*-positions all reacted smoothly with **4a** to afford the corresponding benzylamides **5a–c** in very good isolated yields up to 81%. Electron-donating and -withdrawing groups at the *para*-position were well tolerated (**5d–f**), including halogen substituents that could serve as handles for follow-up functionalisation chemistry such as coupling reactions (**5g**, **5m**, **5n**). Heteroaromatic (**5o**) and aliphatic carboxylic acids (**5j**, **5p**, **5q**) also reacted smoothly under the optimised conditions. As demonstrated by the efficient formation of amide **5q** in 84% yield, the process is tolerant of significant steric bulk at the carboxyl α-position. Finally, to assess the influence of the reaction on the stereochemical integrity of chiral carboxylic acid substrates, the deoxyfluorination was performed on the enantiopure (*S*)-isomer of ibuprofen (er = 99:1). Pleasingly, efficient conversion to the corresponding amide (*S*)-**5l** was observed (yield = 72%) with analysis by chiral HPLC revealing no erosion of the enantiomeric ratio (er = 99:1).

**Scheme 3:**
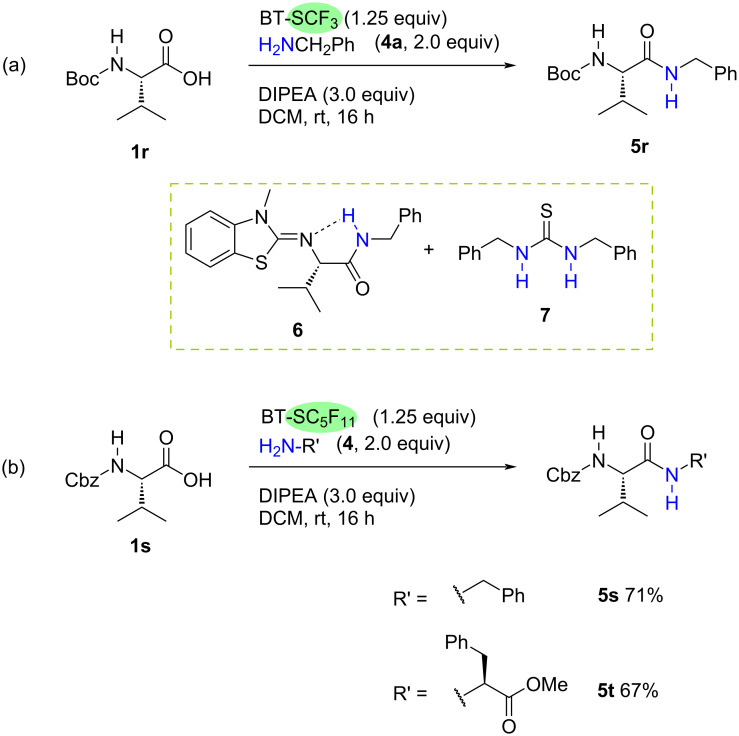
One-pot BT-SCF_3_-mediated deoxygenative coupling of amino acids. Isolated yields after column chromatography.

At this stage, the suitability of BT-SCF_3_-mediated deoxyfluorination for the one-pot formation of peptide linkages between amino acids was investigated (Scheme 3). Treatment of *N*-Boc-valine under the optimised one-pot conditions with benzylamine (**4a**) resulted in the formation of the desired amide product, however, significant by-products were also observed. Careful column chromatography of the crude reaction mixture allowed for the partial isolation and characterisation of the benzothiazolimine species **6** which results from Boc-deprotection and subsequent condensation of the amide product onto the benzothiazolium core. Although the other identified by-product, thiourea **7**, is not derived from the limiting carboxylic acid substrate, it was found to coelute with the amide product, complicating isolation (Scheme 3a). As Boc-deprotection is seemingly feasible under the reaction conditions, to avoid formation of by-product **6**, the process was tested using the *N*-Cbz-valine (**1s**). Moreover, the BT-SCF_3_ reagent was substituted for the longer chain BT-reagent BT-SC_5_F_11_. The use of this benzothiazolium species would avoid the formation of thiocarbonyl difluoride, which is most likely responsible for the generation of thiourea **7**. Pleasingly, under these conditions, amide **5s** was formed smoothly with isolation by column chromatography providing the pure product in 71% yield (Scheme 3b). Furthermore, replacing the benzylamine coupling partner with phenylalanine methyl ester provided dipeptide **5t** in 67% yield (Scheme 3b).

**Scheme 4:**
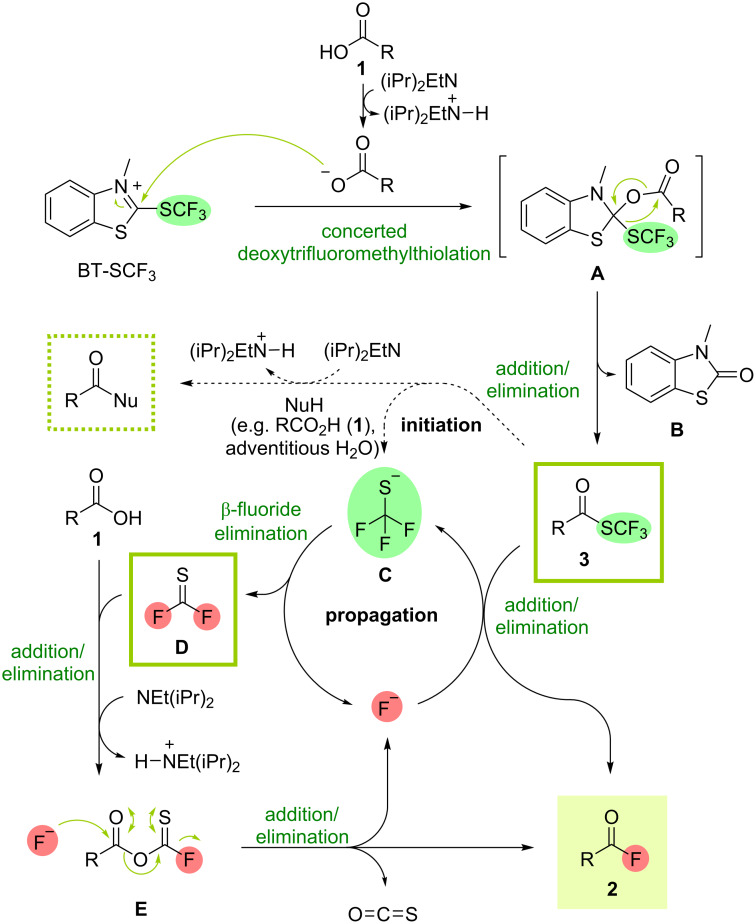
Plausible mechanism for the deoxyfluorination of carboxylic acids with BT-SCF_3_.

**Scheme 5:**
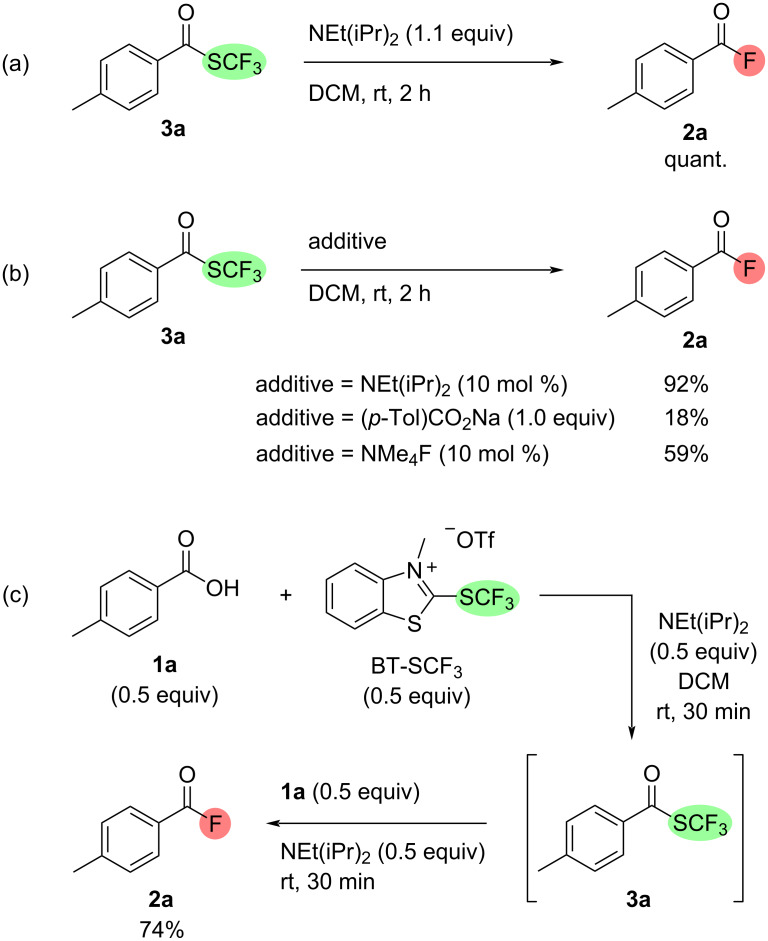
Mechanistic experiments. (a) Conversion of thioester **3a** into acyl fluoride **2a** in the presence of DIPEA. (b) Conversion of thioester **3a** into acyl fluoride **2a** in the presence of carboxylate and fluoride nucleophiles. (c) Two-stage deoxyfluorination reaction using 0.5 equiv of BT-SCF_3_. ^19^F NMR yields using α,α,α-trifluorotoluene as internal standard.

With the scope of the deoxyfluorination process established, our attention turned to an investigation of the reaction mechanism (Scheme 4). As demonstrated in our previous work, reacting BT-SCF_3_ with carboxylic acids **1** under similar conditions provides (trifluoromethyl)thioesters **3** via a concerted deoxytrifluoromethylthiolation process from tetrahedral intermediate **A** affording thiocarbamate by-product **B** [31]. To test whether thioester species could act as intermediates in the formation of acyl fluorides, **3a** was prepared independently and treated with DIPEA (1.1 equiv) in DCM (Scheme 5a). After 1 h at rt, complete consumption of the thioester was observed with acyl fluoride **2a** being obtained as the only product in quantitative ^19^F NMR yield. Conversion of **3** into **2** could result from a self-propagating process initiated by addition of an adventitious nucleophile to the electrophilic thioester. This results in elimination of a (trifluoromethyl)thiolate (^−^SCF_3_) anion (**C**, Scheme 4), which can subsequently undergo β-fluoride elimination, releasing a fluoride anion. Addition of F^−^ to another molecule of thioester **3** thus sets off a chain process, delivering acyl fluoride **2** and regenerating the fluoride nucleophile. A series of experiments conducted with thioester **3a** suggest a number of nucleophiles feasibly present in the reaction mixture can initiate the chain process [34]. Stirring **3a** in the presence of the sodium carboxylate salt of acid **1a** resulted in the formation of **2a** in 18% ^19^F NMR yield while only 10 mol % of tetramethylammonium fluoride (TMAF) provided the acyl fluoride in 59% yield (Scheme 5b). Moreover, efficient conversion of **3a** into **2a** could be achieved using only 10 mol % of DIPEA (92% ^19^F NMR yield, Scheme 5b). This reaction could result from base-assisted nucleophilic attack of adventitious water present in the reaction mixture.

In addition to addition/elimination of fluoride ions to thioesters **3**, a second potential mechanistic pathway exists for the formation of acyl fluorides **2**. Alongside a fluoride ion, β-fluoride elimination from a (trifluoromethyl)thiolate (^−^SCF_3_) anion (**C**) also generates a thiocarbonyl difluoride species **D**. As previously demonstrated by Schoenebeck and co-workers in a deoxyfluorination of carboxylic acids with NMe_4_SCF_3_, this highly electrophilic compound can react with the carboxylic acid in the presence of DIPEA via addition/elimination affording a thioic anhydride species **E** and a fluoride ion [9]. Addition of F**^−^** to the carboxyl carbon followed by fluoride elimination from the resulting thiocarboxylate would provide acyl fluoride **2**, carbonyl sulfide and another fluoride ion. As a result of this pathway, each molecule of the BT-SCF_3_ reagent can in principle lead to the formation of two molecules of acyl fluoride **2**. Indeed, a yield of **2a** above 50% was observed during the optimisation studies using 0.5 equiv of BT-SCF_3_ (Table 1, entry 9). To further investigate the potential for reducing the loading of the deoxyfluorinating reagent, 0.5 equiv of the carboxylic acid substrate **1a** was reacted with 0.5 equiv of both BT-SCF_3_ and DIPEA in DCM for 30 min at rt. ^19^F NMR analysis of the mixture indicated the clean formation of thioester **3a** and the remaining 0.5 equiv of **1a** and 0.5 equiv of DIPEA were then added (Scheme 5c). According to the mechanism shown in Scheme 4, self-propagating conversion of **3a** into **2a**, presumably initiated by a carboxylate nucleophile, would account for half of the acyl fluoride formed with the remaining product resulting from addition of **1** to thiocarbonyl difluoride. After a further 30 minutes at rt, ^19^F NMR analysis of the crude mixture indeed indicated the formation of **2a** in an overall yield of 74%, implying both pathways are feasible and that sub-stoichiometric amounts of BT-SCF_3_ relative to the carboxylic acid can lead to good overall yields of acyl fluorides.

## Conclusion

In conclusion, a practical method for the direct synthesis of acyl fluorides from carboxylic acids using BT-SCF_3_ as a deoxyfluorinating reagent has been developed. In a one-pot process, direct access to various amides was achieved under mild and operationally simple conditions while peptide coupling between two amino acids could be efficiently conducted using the longer-chain perfluoroalkyl reagent BT-SC_5_F_11_. Mechanistic studies revealed that each equivalent of the benzothiazolium reagent can feasibly generate two equivalents of the acyl fluoride with addition/elimination of fluoride to a thioester intermediate and independent deoxyfluorination of a second equivalent of the acid substrate by the released ^−^SCF_3_ anion both operating under the reaction conditions. This allows for the reduction in the loading of BT-SCF_3_ to sub-stoichiometric levels, further increasing the attractiveness of the method.

## Supporting Information

File 1 Experimental procedures, characterisation data of all isolated products as well as copies of NMR spectra for novel compounds.

## Data Availability

The data that supports the findings of this study is available from the corresponding author upon reasonable request.
