# Supplementary material for: Direct synthesis of acyl fluorides from carboxylic acids using benzothiazolium reagents
Source: Beilstein J Org Chem. 2024 Apr 23;20:921–30. doi: 10.3762/bjoc.20.82 (PMC11070953; doi:10.3762/bjoc.20.82)
Supplement: File 1 — Experimental procedures, characterisation data of all isolated products as well as copies of NMR spectra for novel compounds. [file Beilstein_J_Org_Chem-20-921-s001.pdf]

BEILSTEIN JOURNAL OF ORGANIC CHEMISTRY

Supporting Information

for

Direct synthesis of acyl fluorides from carboxylic acids using benzothiazolium reagents

Lilian M. Maas, Alex Haswell, Rory Hughes and Matthew N. Hopkinson

Beilstein J. Org. Chem. **2024,** *20,* 921–930. doi:10.3762/bjoc.20.82

Experimental procedures, characterisation data of all isolated products as well as copies of NMR spectra for novel compounds

License and Terms: This is a supporting information file under the terms of the Creative Commons Attribution License (https://creativecommons.org/licenses/by/4.0). Please note that the reuse, redistribution and reproduction in particular requires that the author(s) and source are credited and that individual graphics may be subject to special legal provisions.
The license is subject to the *Beilstein Journal of Organic Chemistry* terms and conditions: (https://www.beilstein-journals.org/bjoc/terms)

Contents

1 **General information** S1
2 **Optimisation tables** S2
3 **General procedure for the synthesis of acyl fluorides** S3
4 **General procedure for the amide coupling of carboxylic acids with amines** S4
4.1 Analytical data S4
5 **General procedure and analytical data for the coupling of amino acids** S9
5.1 Analytical data S9
6 **Scale-up reaction for the investigation of the side products** S10
7 **NMR Spectra of crude ^{19}F NMR for acid fluoride synthesis** S11
8 **NMR Spectra of literature-unknown product 6** S17
9 **References** S19

1 General Information

All compounds and solvents utilised were purchased from commercial suppliers and used without further treatment, unless specified otherwise. Benzothiazolium reagents were synthesised according to literature known procedures.[1]

Thin-layer chromatography was performed on silica gel coated aluminium plates from TLC Silica gel 60 F_{254} and aluminium oxide 60 F_{254} neutral. The product spots were detected by UV light (254 nm) or as permanganate stains. Flash column chromatography was performed with silica gel 60 M (0.040–0.063 mm, 230–400 mesh).

^{1}H, ^{19}F and ^{13}C NMR spectra were acquired on a Bruker Avance III 300 (300 MHz), Bruker Avance II 400 (400 MHz), Bruker Avance Neo 400 (400 MHz), Bruker Avance III HD 500 (500 MHz), or a Bruker Avance III HD 700 (700 MHz) and analysed on MestReNova 14.1.1. Chemical shifts (δ) are reported in parts per million (ppm) relative to tetramethylsilane (TMS) and coupling constants (*J*) are presented in hertz (Hz). CD_3CN or $CDCl_3$ were used as deuterated solvents and the residual solvent signals were used as references in the ^{1}H and ^{13}C NMR spectra. ^{19}F NMR spectra were not calibrated by an internal reference. ^{19}F NMR yields were measured using α,α,α-trifluorotoluene as an internal standard. The multiplicities are described using the following abbreviations: s = singlet, d = doublet, t = triplet, q = quartet, p = pentet, and m = multiplet.

High-resolution mass spectra were measured with an Agilent (6210 ESI-TOF; 4 µL/min, 1.0 bar, 4 kV). Infrared spectra were measured with a PerkinElmer UATR Two FT-IR Spectrometer. Characteristic absorption bands are displayed in wavenumbers $\tilde{\nu}$ in cm^{-1}.

Chiral normal phase HPLC was performed on a Dionex Ultimate 3000 HPLC unit equipped with UV–vis diode-array detector, fitted with the appropriate Daicel Chiralpak column (dimensions: 0.46 cm ø × 25 cm) along with the corresponding guard column (0.4 cm ø × 1 cm). Wavelengths (λ) are reported in nm, retention times (τ_R) are reported in minutes and solvent flow rates are reported in mL min^{-1}.

2 Optimisation tables

Table S1. Optimisation of the deoxyfluorination of 4-methylbenzoic acid using BT-SCF_3.

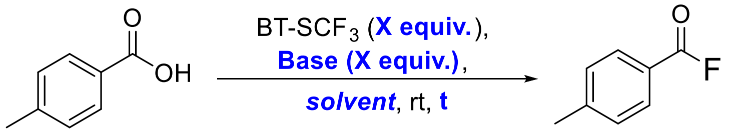

Entry[a]	Reagent equiv.	Base (equiv.)	Solvent	t [h]	Yield Acyl fluoride[b]	Yield Thioester[b]
1	1.25	DIPEA (1.1)	DCM	2	12	60
2	1.25	DIPEA (1.5)	DCM	2	30	45
3	1.25	DIPEA (1.75)	DCM	2	68	17
**4**	**1.25**	**DIPEA (2.0)**	**DCM**	**2**	**Quant.**	**--**
5	1.25	K_2CO_3 (2.0)	DCM	2	7	37
6	1.25	NaH (2.0)	DCM	2	--	30
7	1.25	NEt_3 (2.0)	DCM	2	96	traces
8	1.25	Na_2CO_3 (2.0)	DCM	2	--	26
9	0.5	DIPEA (2.0)	DCM	2	55	--
10	0.75	DIPEA (2.0)	DCM	2	72	--
11	1.0	DIPEA (2.0)	DCM	2	83	--
12	1.5	DIPEA (2.0)	DCM	2	75	--
13	1.25	DIPEA (2.0)	DMF	2	11	--
14	1.25	DIPEA (2.0)	MeCN	2	88	traces
15	1.25	DIPEA (2.0)	EtOAc	2	Quant.	traces
16	1.25	DIPEA (2.0)	THF	2	91	traces
**17**	**1.25**	**DIPEA (2.0)**	**DCM**	**0.5**	**Quant.**	**--**

[a]Reaction procedure: BT-SCF_3, 4-methylbenzoic acid and base were dissolved in the solvent and the reaction mixture was stirred at rt before being concentrated *in vacuo*. [b]Yields calculated by ^{19}F NMR spectroscopy using *α,α,α*-trifluorotoluene as an internal standard.

Table S2. Optimisation of the amide coupling of 4-methylbenzoic acid with benzylamine using BT-SCF_3.

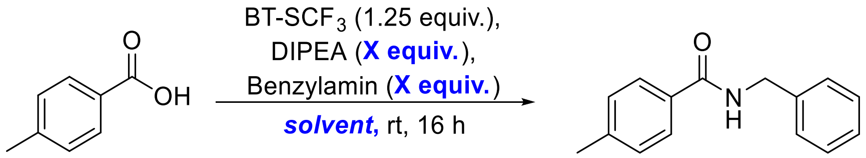

Entry	DIPEA (equiv.)	Benzylamine (equiv.)	Solvent	Yield[c]
1[a]	2.0 then 1.0	1.0	DCM	55
2[a]	2.0 then 2.0	1.0	DCM	50
3[a]	2.0 then 1.0	1.0	MeCN	37
4[a]	2.0 then 1.0	1.0	EtOAc	24
5[a]	2.0 then 1.0	1.5	DCM	83
6[a]	2.0 then 1.0	1.75	DCM	84
7[a]	2.0 then 1.0	2.0	DCM	Quant.
8[b]	3.0	1.0	DCM	43
**9[b]**	**3.0**	**2.0**	**DCM**	**98**
10[b]	2.0	2.0	DCM	90

[a]Reaction procedure: BT-SCF_3, 4-methylbenzoic acid and DIPEA (2.0 equiv.) were dissolved in the solvent and the reaction mixture was stirred at rt for 30 min. Then, DIPEA and benzylamine were added and the mixture was stirred at rt for 16 h before being concentrated *in vacuo*. [b]Reaction procedure: BT-SCF_3, 4-methylbenzoic acid and DIPEA were dissolved in the solvent, then, benzylamine was added and the reaction mixture was stirred at rt for 16 h before being concentrated *in vacuo*. [c]Yields calculated by ^1H NMR spectroscopy using CH_2Br_2 as an internal standard.

3 General procedure for the synthesis of acyl fluorides

To a solution of BT-SCF_3 (1.25 equiv, 0.63 mmol, 250 mg) in DCM (5 mL) was added benzoic acid derivatives **1** (1.0 equiv, 0.5 mmol) and DIPEA (2.0 equiv, 1.0 mmol, 175 µL). The resulting mixture was stirred for 2 h at rt. The solvent was removed under reduced pressure, $CDCl_3$ (0.65 mL) and α,α,α-trifluorotoluene (0.5 mmol, 63 µL) were added and the crude NMR yields were determined from ^{19}F NMR spectra.

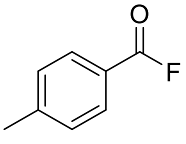

2a (quant.)

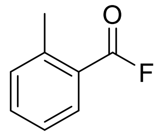

2b (89%)

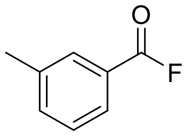

2c (quant.)

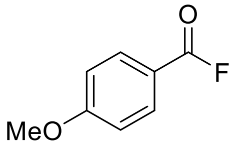

2d (85%)

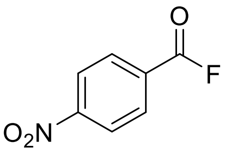

2e (95%)

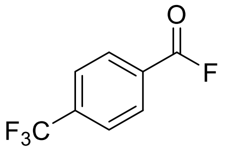

2f (75%)

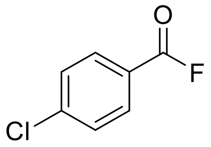

2g (quant.)

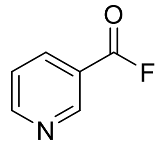

2h (90%)

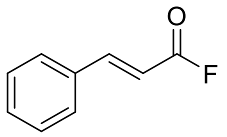

2i (80%)

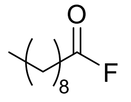

2j (89%)

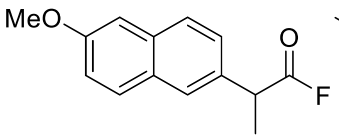

2k (97%)

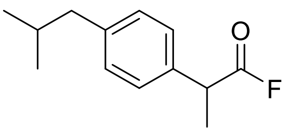

2l (quant.)

Crude **^{19}F NMR (282 MHz, $CDCl_3$)** Signals:

**2a**	δ [ppm] = 17.3.	**2h**	δ [ppm] = 20.6.
**2b**	δ [ppm] = 29.1.	**2i**	δ [ppm] = 25.4 (d, J = 7.4 Hz).
**2c**	δ [ppm] = 18.1.	**2j**	δ [ppm] = 45.3.
**2d**	δ [ppm] = 15.8.	**2k**	δ [ppm] = 39.3.
**2e**	δ [ppm] = 21.3.	**2l**	δ [ppm] = 39.1.
**2f**	δ [ppm] = 19.9 (F), –63.5 (CF_3).		
**2g**	δ [ppm] = 18.3.		

The analytical data are in agreement with the literature data.[2]

4 General procedure for the amide coupling of carboxylic acids with amines

To a solution of BT-SCF_3 (1.25 equiv, 0.63 mmol, 250 mg) in DCM (5 mL) was added benzoic acid derivatives **1** (1.0 equiv, 0.5 mmol) and DIPEA (3.0 equiv, 1.5 mmol, 262 µL). Then, benzylamine **4a** (2.0 equiv, 1.0 mmol, 109 µL) was added and the resulting mixture was stirred overnight at rt. The solvent was removed under reduced pressure and the products **5** were purified by flash chromatography on a silica gel column.

4.1 Analytical data

N-Benzyl-4-methylbenzamide (**5a**)

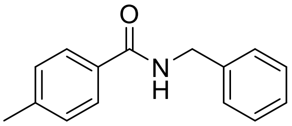

Amide **5a** (90 mg, 0.40 mmol, 80%) was obtained from 4-methylbenzoic acid after flash column chromatography (petroleum ether / EtOAc, 5:1) as a pale-yellow solid.

^{1}H NMR (400 MHz, $CDCl_3$) δ [ppm] = 7.65 (d, *J* = 8.2 Hz, 2H), 7.30 – 7.15 (m, 5H), 7.13 (d, *J* = 7.9 Hz, 2H), 6.81 (bs, 1H), 4.53 (d, *J* = 5.8 Hz, 2H), 2.33 (s, 3H). **^{13}C NMR** (75 MHz, $CDCl_3$) δ [ppm] = 167.4, 142.0, 138.5, 131.6, 129.3, 128.8, 128.0, 127.6, 127.1, 44.1, 21.5.
The characterization data agree with the literature values.[3]

N-Benzyl-2-methylbenzamide (**5b**)

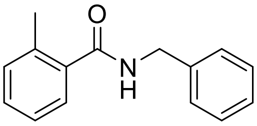

Amide **5b** (91 mg, 0.40 mmol, 81%) was obtained from 2-methylbenzoic acid after flash column chromatography (petroleum ether / EtOAc, 5:1→3:1) as a pale-yellow solid.

^{1}H NMR (300 MHz, $CDCl_3$) δ [ppm] = δ 7.32 – 7.17 (m, 7H), 7.15 – 7.05 (m, 2H), 6.16 (bs, 1H), 4.51 (d, *J* = 5.9 Hz, 2H), 2.36 (s, 3H). **^{13}C NMR** (75 MHz, $CDCl_3$) δ [ppm] = 170.0, 138.3, 136.3, 136.2, 131.1, 130.0, 128.8, 127.9, 127.6, 126.8, 125.8, 43.9, 19.9.
The characterization data agree with the literature values.[4]

N-Benzyl-3-methylbenzamide (**5c**)

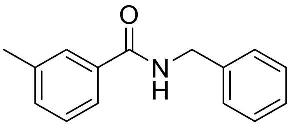

Amide **5c** (80 mg, 0.36 mmol, 71%) was obtained from 3-methylbenzoic acid after flash column chromatography (petroleum ether / EtOAc, 5:1→3:1) as a pale-yellow solid.

^{1}H NMR (300 MHz, $CDCl_3$) δ [ppm] = 7.62 (s, 1H), 7.59 – 7.53 (m, 1H), 7.36 – 7.32 (m, 4H), 7.30 – 7.26 (m, 3H), 6.50 (s, 1H), 4.63 (d, *J* = 5.7 Hz, 2H), 2.38 (s, 3H). **^{13}C NMR** (75 MHz, $CDCl_3$) δ [ppm] = 167.7, 138.6, 138.4, 134.5, 132.4, 128.9, 128.6, 128.0, 127.9, 127.7, 124.0, 44.2, 21.5.
The characterization data agree with the literature values.[4]

N-Benzyl-4-methoxybenzamide (**5d**)

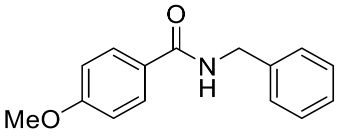

Amide **5d** (98 mg, 0.4 mmol, 80%) was obtained from 4-methoxybenzoic acid after flash column chromatography (petroleum ether / EtOAc, 2:1→1:1) as a pale-yellow solid.

^{1}**H NMR** (400 MHz, $CDCl_3$) δ [ppm] = 7.71 (d, *J* = 8.8 Hz, 2H), 7.32 – 7.17 (m, 5H), 6.82 (d, *J* = 8.8 Hz, 2H), 6.67 (bs, 1H), 4.53 (d, *J* = 5.7 Hz, 2H), 3.76 (s, 3H). ^{13}**C NMR** (75 MHz, $CDCl_3$) δ [ppm] = 167.0, 162.3, 138.6, 128.9, 128.8, 128.0, 127.6, 126.8, 113.9, 55.5, 44.1.
The characterization data agree with the literature values.[3]

N-benzyl-4-nitrobenzamide (**5e**)

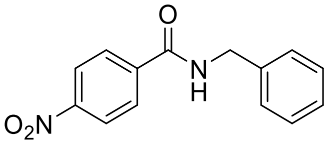

Amide **5e** (89 mg, 0.35 mmol, 70%) was obtained from 4-nitrobenzoic acid after flash column chromatography (petroleum ether / EtOAc, 2:1) as a pale-yellow solid.

^{1}**H NMR** (300 MHz, $CDCl_3$) δ [ppm] = 8.28 (d, *J* = 8.9 Hz, 2H), 7.95 (d, *J* = 8.8 Hz, 2H), 7.43 – 7.29 (m, 5H), 6.49 (bs, 1H), 4.67 (d, *J* = 5.6 Hz, 2H). ^{13}**C NMR** (75 MHz, $CDCl_3$) δ [ppm] = 165.5, 149.7, 140.0, 137.6, 129.1, 128.3, 128.1, 128.1, 124.0, 44.6. **IR (ATR)**: $\tilde{\nu}$ [cm^{-1}] = 3277, 3035, 2925, 2851, 1944, 1812, 1629, 1596, 1533, 1511, 1484, 1453, 1463, 1362, 1344, 1317, 1281, 1221, 1178, 1147, 1077, 1060, 1031, 1012, 988, 959, 914, 902, 872, 853, 796, 782, 752, 725, 663, 622, 584, 548, 515, 504, 465, 424, 410.
The characterization data agree with the literature values.[5, 3b]

N-Benzyl-4-(trifluoromethyl)benzamide **(5f)**

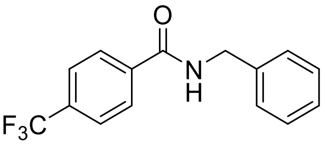

Amide **5f** (113 mg, 0.4 mmol, 81%) was obtained from 4-(trifluoromethyl)benzoic acid after flash column chromatography (petroleum ether / EtOAc, 5:1→3:1) as a pale-yellow solid.

^{1}**H NMR** (300 MHz, $CDCl_3$) δ [ppm] = 7.89 (d, *J* = 8.0 Hz, 2H), 7.68 (d, *J* = 8.1 Hz, 2H), 7.44 – 7.28 (m, 5H), 6.51 (bs, 1H), 4.65 (d, *J* = 5.6 Hz, 2H). ^{19}**F NMR** (282 MHz, $CDCl_3$) δ [ppm] = -62.96. ^{13}**C NMR** (75 MHz, $CDCl_3$) δ [ppm] = 166.2, 137.9, 137.8, 133.5 (q, *J* = 33 Hz),129.0, 128.1, 128.0, 127.6, 125.8 (q, *J* = 4 Hz), 123.8 (q, *J* = 273 Hz), 44.5.
The characterization data agree with the literature values.[6]

N-Benzyl-4-chlorobenzamide (**5g**)

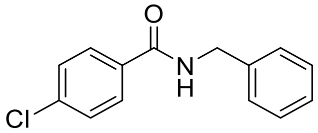

Amide **5g** (105 mg, 0.43 mmol, 85%) was obtained from 4-chlorobenzoic acid after flash column chromatography (petroleum ether / EtOAc, 5:1) as a pale-yellow solid.

^{1}**H NMR** (300 MHz, $CDCl_3$) δ [ppm] = 7.77 – 7.68 (m, 2H), 7.44 – 7.27 (m, 7H), 6.47 (bs, 1H), 4.62 (d, *J* = 5.7 Hz, 2H). ^{13}**C NMR** (75 MHz, $CDCl_3$) δ [ppm] = 166.4, 138.1, 137.9, 132.9, 129.0, 128.5, 128.1, 127.9, 44.4.
The characterization data agree with the literature values.[7]

N-Benzyl-4-bromobenzamide (**5m**)

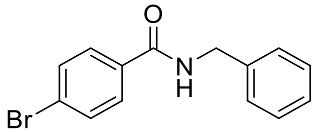

Amide **5m** (145 mg, 0.49 mmol, 98%) was obtained from 4-bromobenzoic acid after flash column chromatography (petroleum ether / EtOAc, 5:1) as a pale-yellow solid.

^{1}**H NMR** (300 MHz, $CDCl_3$) δ [ppm] = 7.65 (d, *J* = 8.4 Hz, 2H), 7.54 (d, *J* = 8.6 Hz, 2H), 7.41 – 7.27 (m, 5H), 6.50 (bs, 1H), 4.61 (d, *J* = 5.7 Hz, 2H). ^{13}**C NMR** (75 MHz, $CDCl_3$) δ [ppm] = 166.5, 138.0, 133.3, 131.9, 129.0, 128.7, 128.1, 127.9, 126.4, 44.3.
The characterization data agree with the literature values.[8]

N-Benzyl-4-iodobenzamide (**5n**)

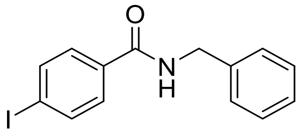

Amide **5n** (142 mg, 0.42 mmol, 84%) was obtained from 4-iodobenzoic acid after flash column chromatography (petroleum ether / EtOAc, 5:1→3:1) as a pale-yellow solid.

^{1}**H NMR** (300 MHz, $CDCl_3$) δ [ppm] = 7.77 (d, *J* = 8.6 Hz, 2H), 7.50 (d, *J* = 8.5 Hz, 2H), 7.43 – 7.27 (m, 5H), 6.45 (bs, 1H), 4.62 (d, *J* = 5.6 Hz, 2H). ^{13}**C NMR** (75 MHz, $CDCl_3$) δ [ppm] = 166.7, 138.0, 137.9, 133.9, 129.0, 128.7, 128.1, 127.9, 98.6, 44.4.
The characterization data agree with the literature values.[8]

N-Benzyl-6-chloronicotinamide (**5o**)

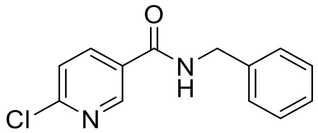

Amide **5o** (92 mg, 0.37 mmol, 74%) was obtained from 6-chloronicotinic acid after flash column chromatography (petroleum ether / EtOAc, 3:1) as a pale-yellow solid.

^{1}**H NMR** (400 MHz, $CDCl_3$) δ [ppm] = 8.69 (d, *J* = 2.6 Hz, 1H), 8.04 (dd, *J* = 8.3, 2.6 Hz, 1H), 7.38 – 7.13 (m, 6H), 7.13 (dd, *J* = 7.7, 3.1 Hz, 2H), 6.61 (bs, 1H), 4.58 (d, *J* = 5.6 Hz, 2H). ^{13}**C NMR** (101 MHz, $CDCl_3$) δ [ppm] = 164.6, 154.4, 148.1, 138.1, 137.6, 129.0, 128.4, 128.1, 128.0, 124.5, 44.4.
The characterization data agree with the literature values.[9]

N-Benzyldecanamide (**5j**)

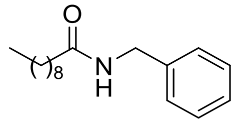

Amide **5j** (119 mg, 0.46 mmol, 91%) was obtained from decanoic acid after flash column chromatography (petroleum ether / EtOAc, 3:1) as a pale-yellow solid.

^{1}**H NMR** (400 MHz, $CDCl_3$) δ [ppm] = 7.35 – 7.21 (m, 5H), 6.26 (bs, 1H), 4.39 (d, *J* = 5.8 Hz, 2H), 2.19 (t, *J* = 7.6 Hz, 2H), 1.67 – 1.58 (m, 2H), 1.33 – 1.24 (s, 12H), 0.89 (t, *J* = 6.9 Hz, 3H). ^{13}**C NMR** (101 MHz, $CDCl_3$) δ [ppm] = 173.3, 138.6, 128.7, 127.8, 127.4, 43.5, 36.7, 31.9, 29.5, 29.43, 29.39, 29.32, 25.9, 22.7, 14.1.
The characterization data agree with the literature values.[10]

N-Benzyl-2-phenylpropanamide (**5p**)

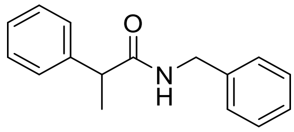

Amide **5p** (111 mg, 0.46 mmol, 93%) was obtained from 2-phenylpropanoic acid after flash column chromatography (petroleum ether / EtOAc, 3:1) as a pale-yellow solid.

^{1}**H NMR** (400 MHz, $CDCl_3$) δ [ppm] = 7.33 – 7.13 (m, 8H), 7.10 – 7.05 (m, 2H), 5.63 (bs, 1H), 4.41 – 4.26 (m, 2H), 3.55 (q, *J* = 7.2 Hz, 1H), 1.50 (d, *J* = 7.2 Hz, 3H). ^{13}**C NMR** (101 MHz, $CDCl_3$) δ [ppm] = 174.2, 141.4, 138.4, 129.1, 128.7, 127.8, 127.6, 127.49, 127.45, 47.3, 43.7, 18.7.
The characterization data agree with the literature values.[11]

N-Benzyl-2-methyl-2-phenylpropanamide (**5q**)

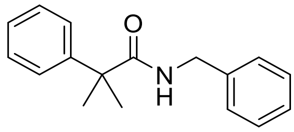

Amide **5q** (107 mg, 0.42 mmol, 84%) was obtained from 2-methyl-2-phenylpropanoic acid after flash column chromatography (petroleum ether / EtOAc, 3:1) as a pale-yellow solid.

^{1}**H NMR** (400 MHz, $CDCl_3$) δ [ppm] = 7.41 – 7.30 (m, 4H), 7.30 – 7.20 (m, 4H), 7.13 – 7.09 (m, 2H), 5.45 (s, 1H), 4.37 (d, *J* = 5.8 Hz, 2H), 1.61 (s, 6H). ^{13}**C NMR** (101 MHz, $CDCl_3$) δ [ppm] = 177.5, 145.2, 138.6, 128.9, 128.7, 127.5, 127.4, 127.2, 126.6, 47.2, 43.8, 27.2.
The characterization data agree with the literature values.[12]

(*S*)-*N*-Benzyl-2-(4-isobutylphenyl)propenamide (**5l**)

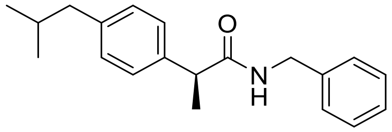

Amide **5l** (107 mg, 0.36 mmol, 72%) was obtained from (2S)-2-(4-Isobutylphenyl)propanoic acid after flash column chromatography (petroleum ether / EtOAc, 3:1) as a white solid.

^{1}H NMR (300 MHz, $CDCl_3$) δ [ppm] = 7.27 – 7.12 (m, 5H), 7.12 – 7.03 (m, 4H), 5.67 (bs, 1H), 4.34 (d, *J* = 5.9 Hz, 2H), 3.54 (q, *J* = 7.2 Hz, 1H), 2.41 (d, *J* = 7.1 Hz, 2H), 1.90 – 1.72 (m, 1H), 1.51 (d, *J* = 7.2 Hz, 3H), 0.86 (d, *J* = 6.6 Hz, 6H). **^{13}C NMR** (75 MHz, $CDCl_3$) δ [ppm] = 174.5, 140.9, 138.6, 138.5, 129.8, 128.7, 127.5, 127.4, 46.9, 45.1, 43.6, 30.3, 22.5, 18.6.*

*Note: One carbon peak could not be observed, due to overlapping signals.

Chiral HPLC: DAICEL Chiralpak IC column with guard; solvent ratio = 90:10, *n*-hexane:^{i}PrOH. Temperature = 25 °C. Flow rate = 1 mL/min, λ = 210 nm, τ_{ret} = 15.1 min and 18.7 min.

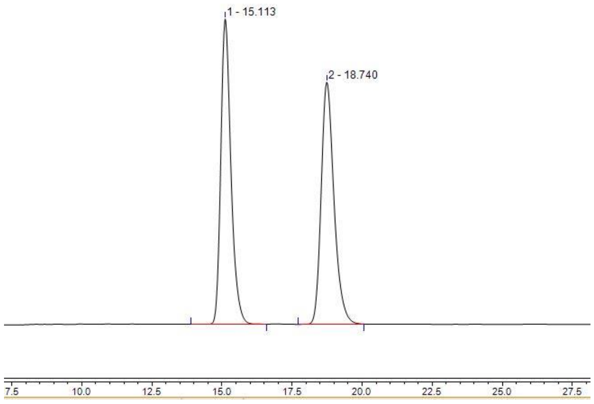

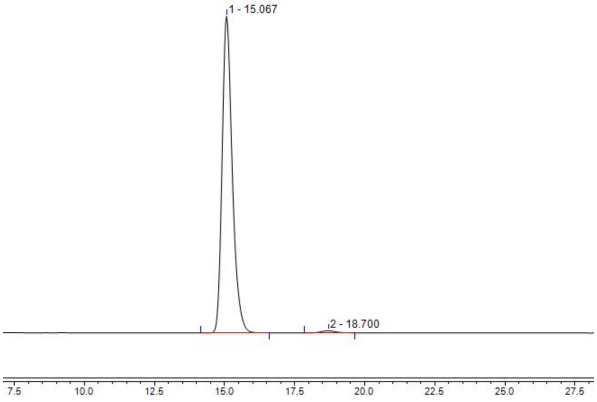

Ret.Time min	Amount n.a.	Rel.Area %	Area mAU*min	Height mAU
15.113	n.a.	49.89	174.2813	415.48
18.740	n.a.	50.11	175.0514	329.64

Ret.Time min	Amount n.a.	Rel.Area %	Area mAU*min	Height mAU
15.067	n.a.	99.09	147.9451	351.35
18.700	n.a.	0.91	1.3633	2.36

The characterization data agree with the literature values.[13]

5 General procedure and analytical data for the coupling of amino acids

To a solution of BT-SC_5F_{11} (1.25 equiv, 0.63 mmol, 375 mg) in DCM (5 mL) was added Cbz-valine **1s** (1.0 equiv, 0.5 mmol, 126 mg) and DIPEA (3.0–5.0 equiv). Then, amine **4** (2.0 equiv, 1.0 mmol) was added and the resulting mixture was stirred overnight at rt. The solvent was removed under reduced pressure and the products **5s-t** were purified by flash chromatography on a silica gel column.

5.1 Analytical data

Benzyl (S)-(1-(benzylamino)-3-methyl-1-oxobutan-2-yl)carbamate (**5s**)

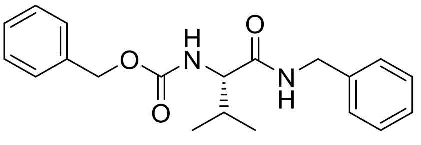

Amide **5s** (121 mg, 0.36 mmol, 71%) was obtained using benzylamine (2.0 equiv, 109 µL) and DIPEA (3.0 equiv, 1.5 mmol, 262 µL), after flash column chromatography (petroleum ether / EtOAc, 3:1) as a white solid.

^{1}H NMR (300 MHz, $CDCl_3$) δ [ppm] = 7.36 – 7.15 (m, 10H), 6.49 (bs, 1H), 5.42 (d, *J* = 7.7 Hz, 1H), 5.05 – 4.89 (m, 2H), 4.37 (dd, *J* = 14.8, 5.8 Hz, 2H), 4.04 – 3.93 (m, 1H), 2.16 – 2.01 (m, 2H), 0.92 (d, *J* = 6.8 Hz, 3H), 0.88 (d, *J* = 6.8 Hz, 3H). **^{13}C NMR** (75 MHz, $CDCl_3$) δ [ppm] = 171.3, 156.6, 138.0, 136.3, 128.8, 128.7, 128.3, 128.1, 127.9, 127.7, 67.2, 60.7, 43.7, 31.2, 19.4, 18.0.
The characterization data agree with the literature values.[14]

Methyl ((benzyloxy)carbonyl)-L-valyl-L-phenylalaninate (**5t**)

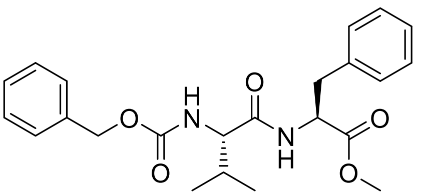

Amide **5t** (139 mg, 0.34 mmol, 67%) was obtained using methyl L-phenylalaninate hydrochloride (216 mg) and DIPEA (5.0 equiv, 2.5 mmol, 435 µL) after flash column chromatography (petroleum ether / EtOAc, 3:1) as a white solid.

^{1}H NMR (300 MHz, $CDCl_3$) δ [ppm] = 7.43 – 7.07 (m, 10H), 6.73 (d, *J* = 8.0 Hz, 1H), 5.58 (d, *J* = 9.0 Hz, 1H), 5.20 – 5.01 (m, 2H), 4.93 (m, 1H), 4.19 – 4.08 (m, 1H), 3.72 (s, 3H), 3.19 – 3.05 (m, 2H), 2.10 (h, *J* = 6.9 Hz, 1H), 0.97 (d, *J* = 6.7 Hz, 3H), 0.92 (d, *J* = 6.8 Hz, 3H). **^{13}C NMR** (75 MHz, $CDCl_3$) δ [ppm] = 171.8, 171.11, 156.4, 136.4, 135.7, 129.3, 128.6, 128.5, 128.2, 128.0, 127.2, 67.0, 60.2, 53.2, 52.3, 38.0, 31.3, 19.1, 17.8.
The characterization data agree with the literature values.[15]

6 Scale-up reaction for the investigation of the side products

To a solution of BT-SCF_3 (1.25 equiv, 6.25 mmol, 2.50 g) in DCM (50 mL) was added Boc-valine **1r** (1.0 equiv, 5.0 mmol, 1.09 g) and DIPEA (3.0 equiv, 15.0 mmol, 1.31 mL). Then, benzylamine (**4a**, 2.0 equiv, 10.0 mmol, 1.09 mL) was added and the resulting mixture was stirred overnight at rt. The solvent was removed under reduced pressure and product **6** and **7** were purified by flash chromatography on a silica gel column.

6.1 Analytical data

(S,Z)-*N*-Benzyl-3-methyl-2-((3-methylbenzo[d]thiazol-2(3*H*)-ylidene)amino)butanamide (**6**)

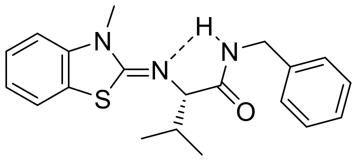

Side product **6** was obtained after flash column chromatography (petroleum ether / EtOAc, 3:1) as a pale-yellow oil.

^{1}**H NMR** (400 MHz, $CDCl_3$) δ [ppm] = 7.33 – 7.18 (m, 7H), 7.03 (m, 1H), 6.99 (d, *J* = 7.5 Hz, 1H), 6.86 (d, *J* = 8.1 Hz, 1H), 4.55 (dd, *J* = 15.0, 6.3 Hz, 1H), 4.38 (dd, *J* = 15.1, 5.7 Hz, 1H), 3.58 (d, *J* = 3.6 Hz, 1H), 3.40 (s, 3H), 2.38 (m, 1H), 0.97 (d, *J* = 6.8 Hz, 3H), 0.94 (d, *J* = 6.9 Hz, 3H).^{13}**C NMR** (101 MHz, $CDCl_3$) δ [ppm] = 173.1, 157.8, 140.9, 138.8, 128.7, 127.6, 127.4, 126.5, 122.3, 122.2, 121.4, 108.8, 74.1, 43.1, 33.4, 30.4, 20.0, 17.7. **IR (ATR)**: ṽ [cm^{-1}]: 3378, 3062, 3030, 2960, 2928, 2871, 1717, 1663, 1629, 1585, 1509, 1480, 1456, 1418, 1383, 1362, 1350, 1305, 1323, 1252, 1160, 1135, 1027, 960, 923, 851, 742, 716, 699, 610, 542, 517, 495, 479. **HRMS (ESI-TOF)** calculated for $[C_{20}H_{23}N_3OS]^+$ ($[M]^+$): 354.1635, measured: 354.1624.

1,3-Dibenzylthiourea (**7**)

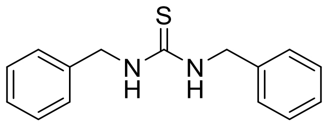

Side product **7** was obtained after flash column chromatography (petroleum ether / EtOAc, 3:1) as a pale-yellow solid.

^{1}**H NMR** (300 MHz, $CDCl_3$): δ [ppm] = 7.35-7.28 (m, 6H), 7.26 – 7.21 (m, 4H), 6.00 (s, 2H), 4.64 (d, J = 5.3 Hz, 4H). The characterization data agree with the literature values.[16]

7 NMR Spectra of crude ^{19}F NMR for acid fluoride synthesis

Crude ^{19}F NMR (282 MHz in $CDCl_3$), 298 K

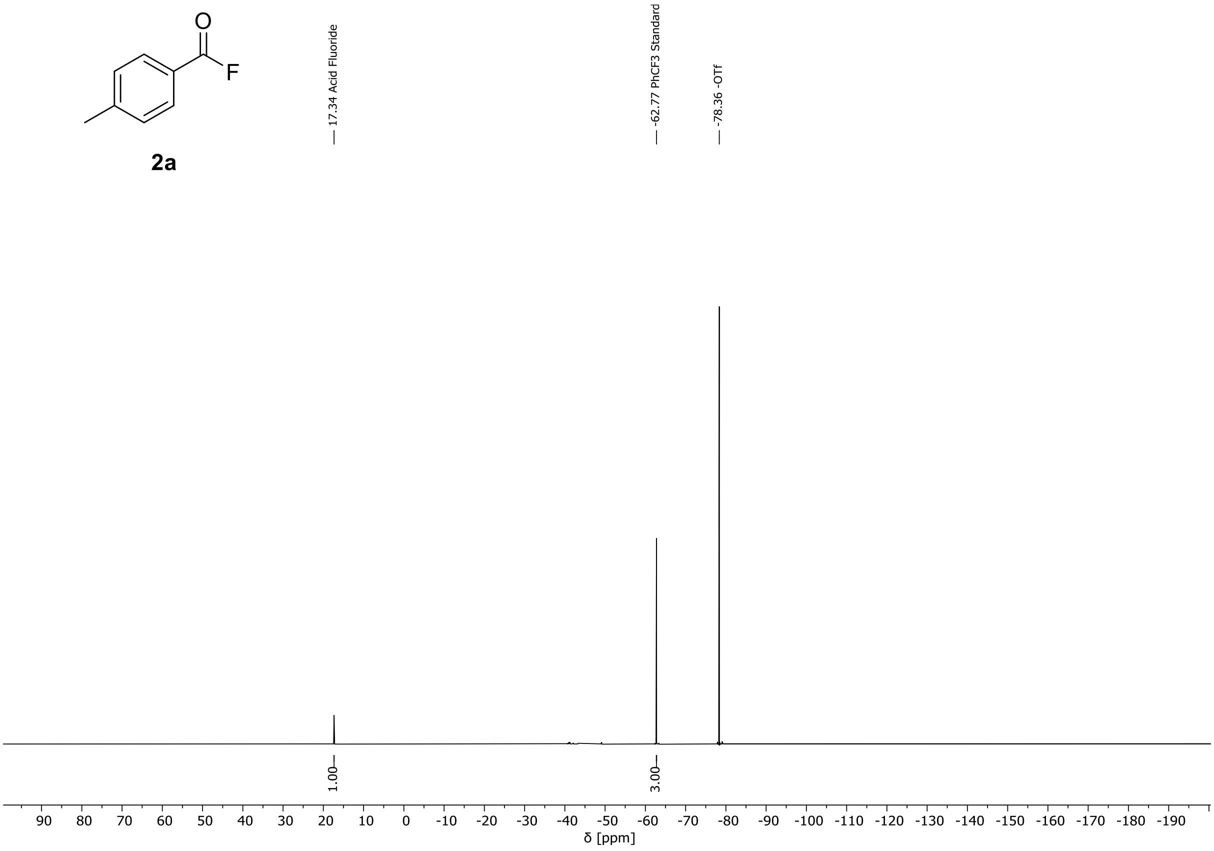

2a

Crude ^{19}F NMR (282 MHz in $CDCl_3$), 298 K

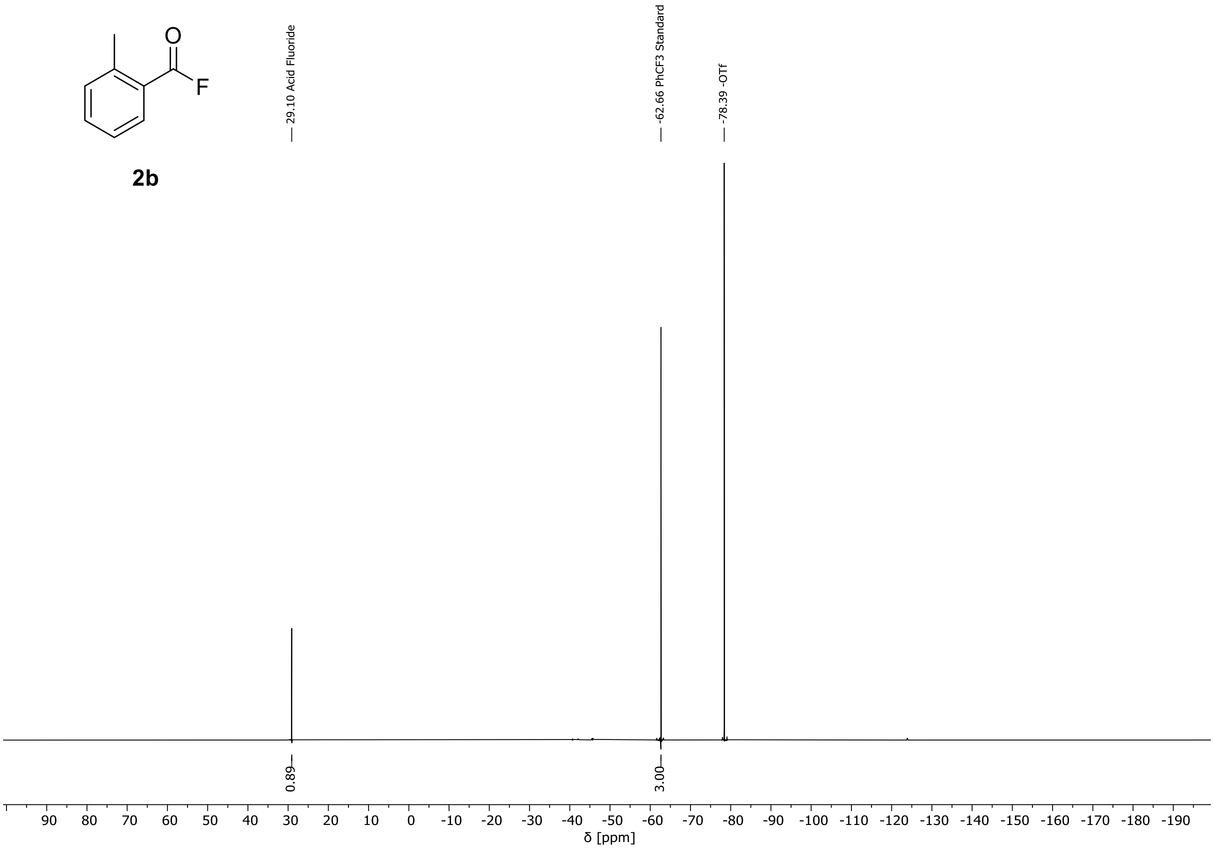

2b

Crude ^{19}F NMR (282 MHz in $CDCl_3$), 298 K

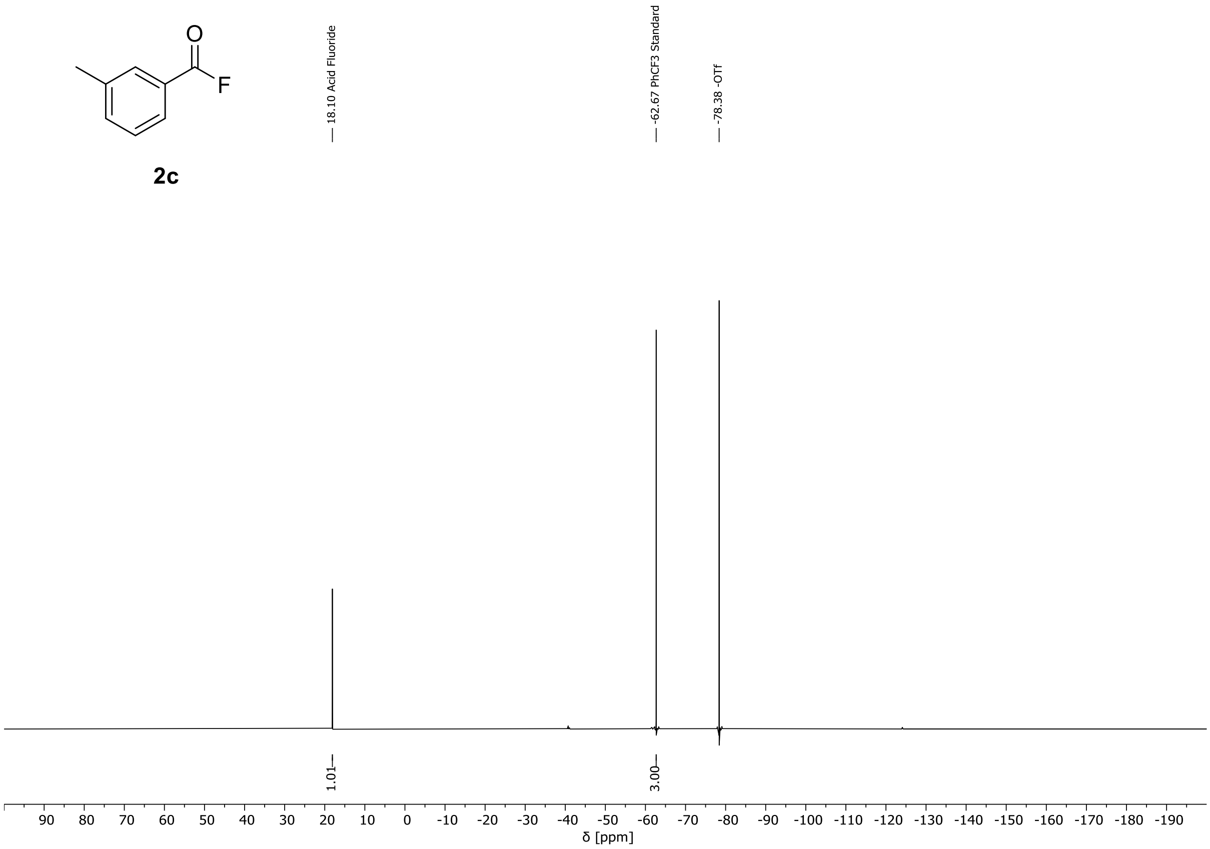

2c

Crude ^{19}F NMR (282 MHz in $CDCl_3$), 298 K

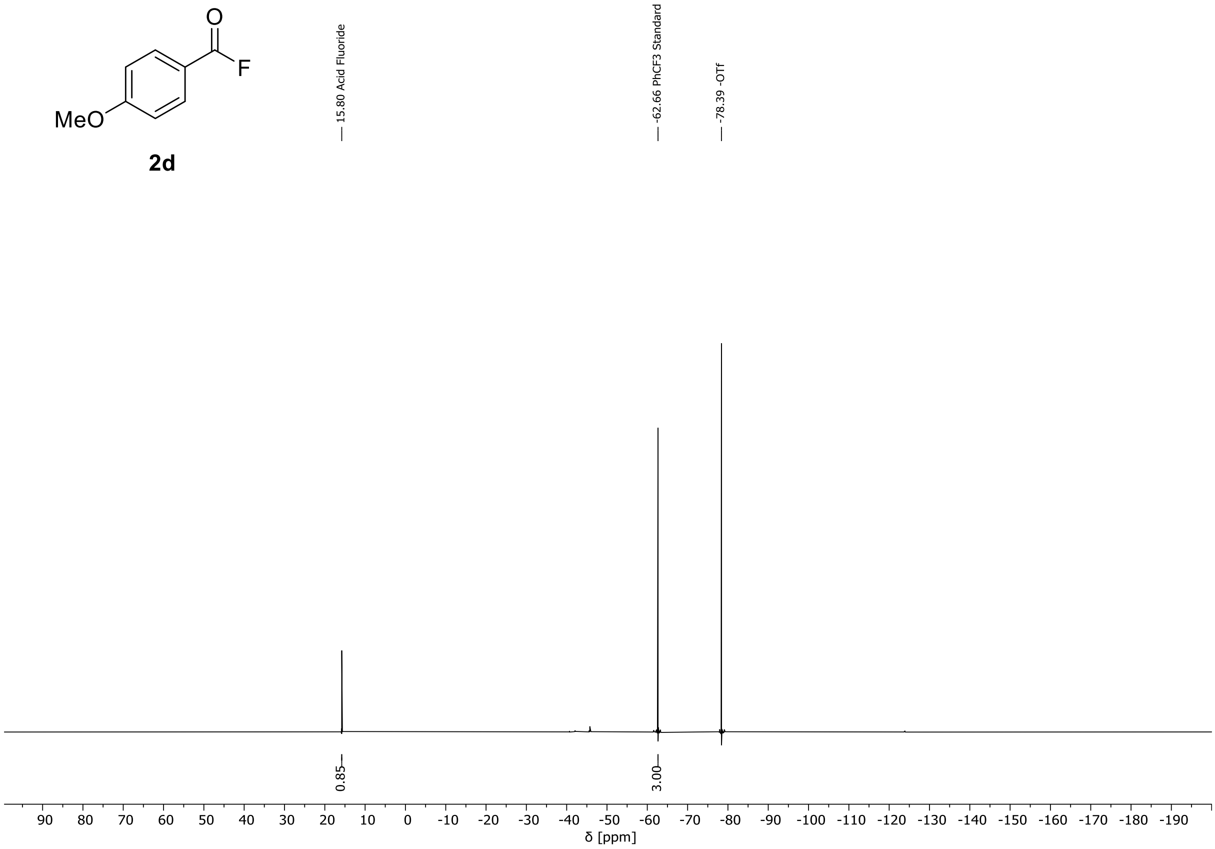

2d

Crude ^{19}F NMR (282 MHz in $CDCl_3$), 298 K

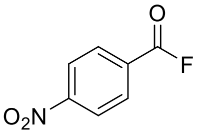

2e

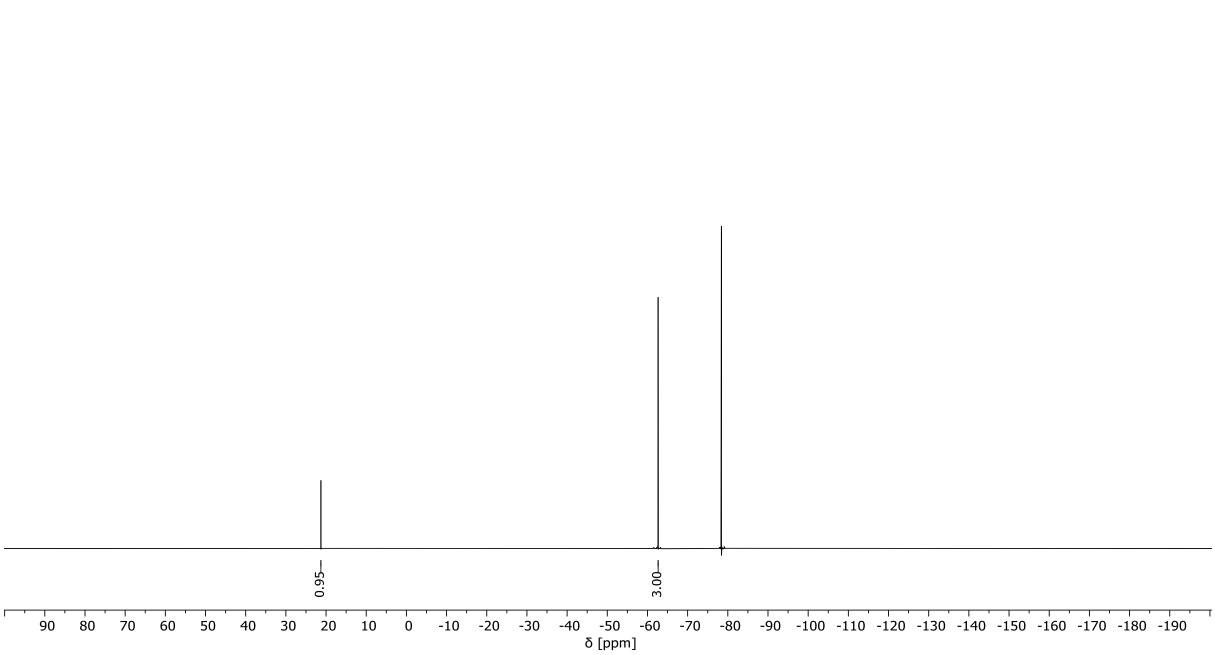

Crude ^{19}F NMR (282 MHz in $CDCl_3$), 298 K

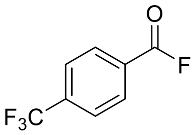

2f

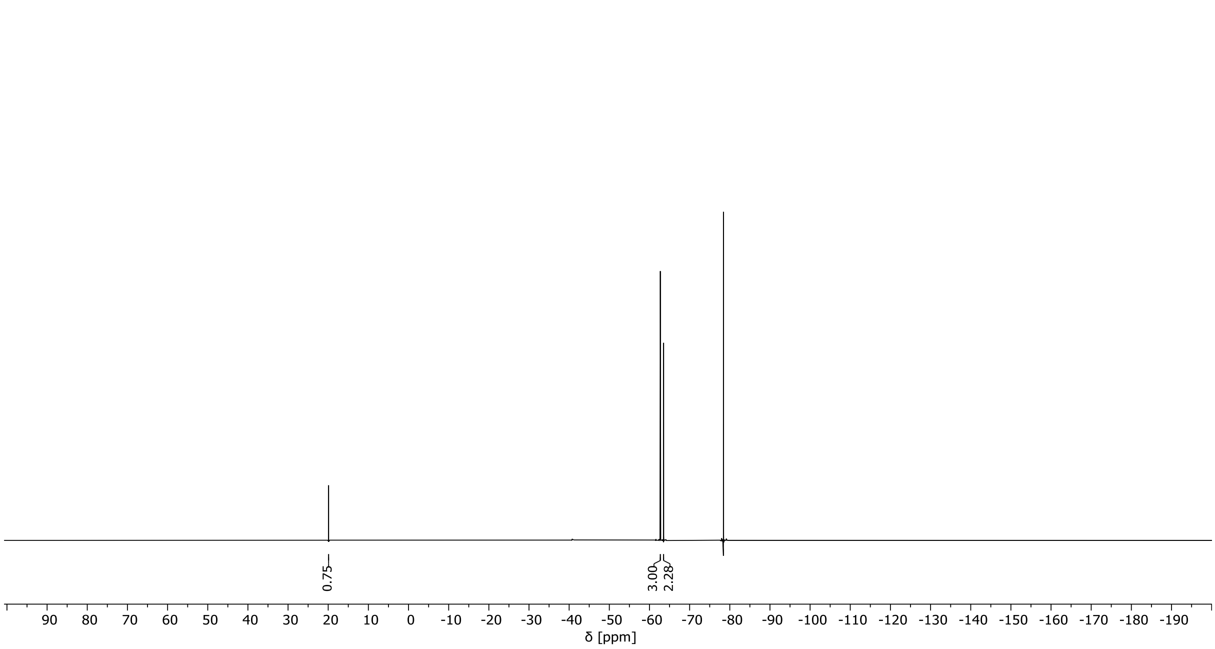

Crude ^{19}F NMR (282 MHz in $CDCl_3$), 298 K

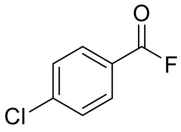

2g

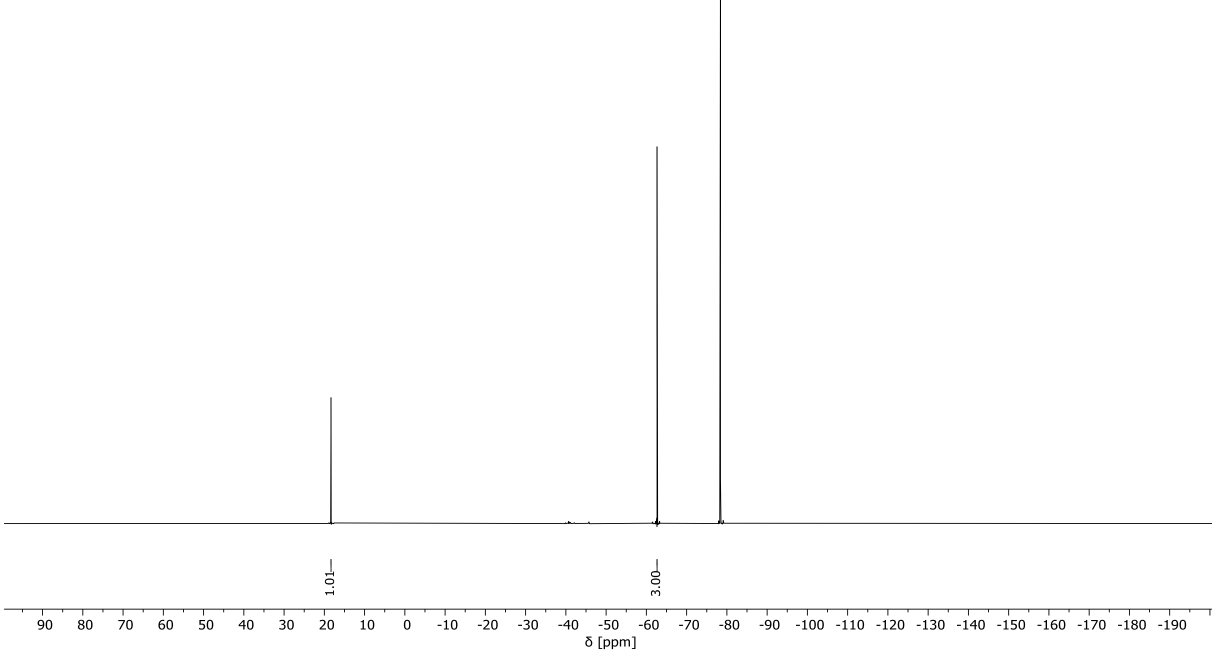

Crude ^{19}F NMR (282 MHz in $CDCl_3$), 298 K

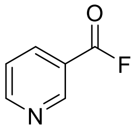

2h

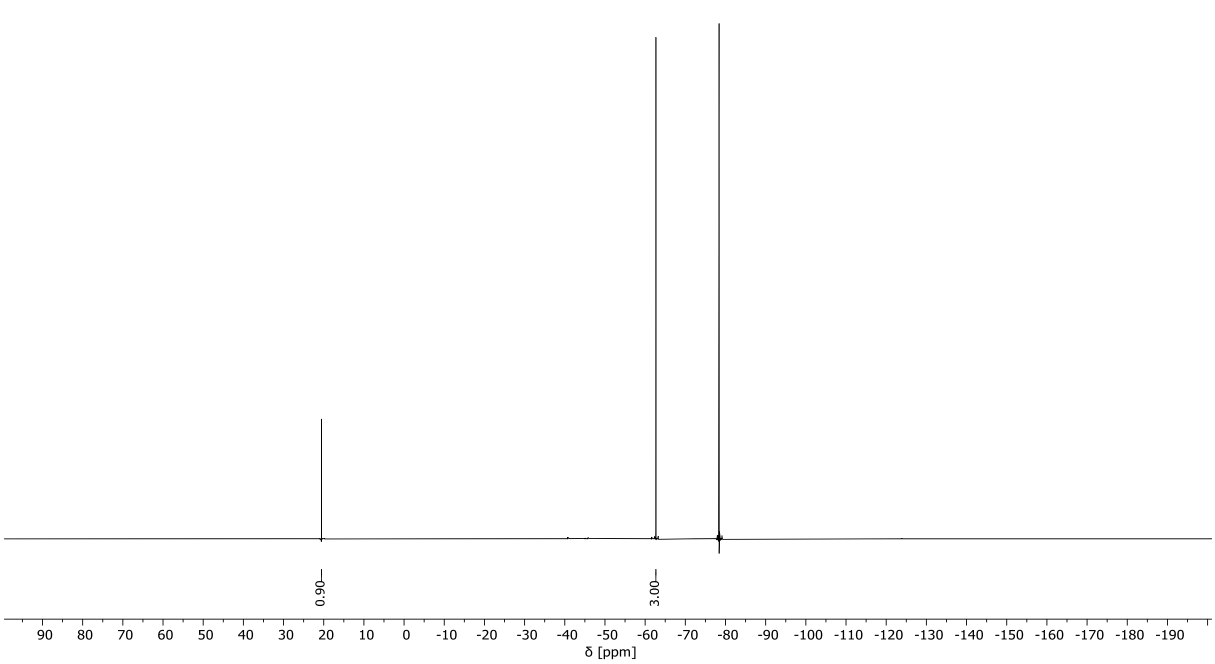

Crude ^{19}F NMR (282 MHz in $CDCl_3$), 298 K

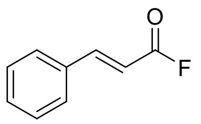

2i

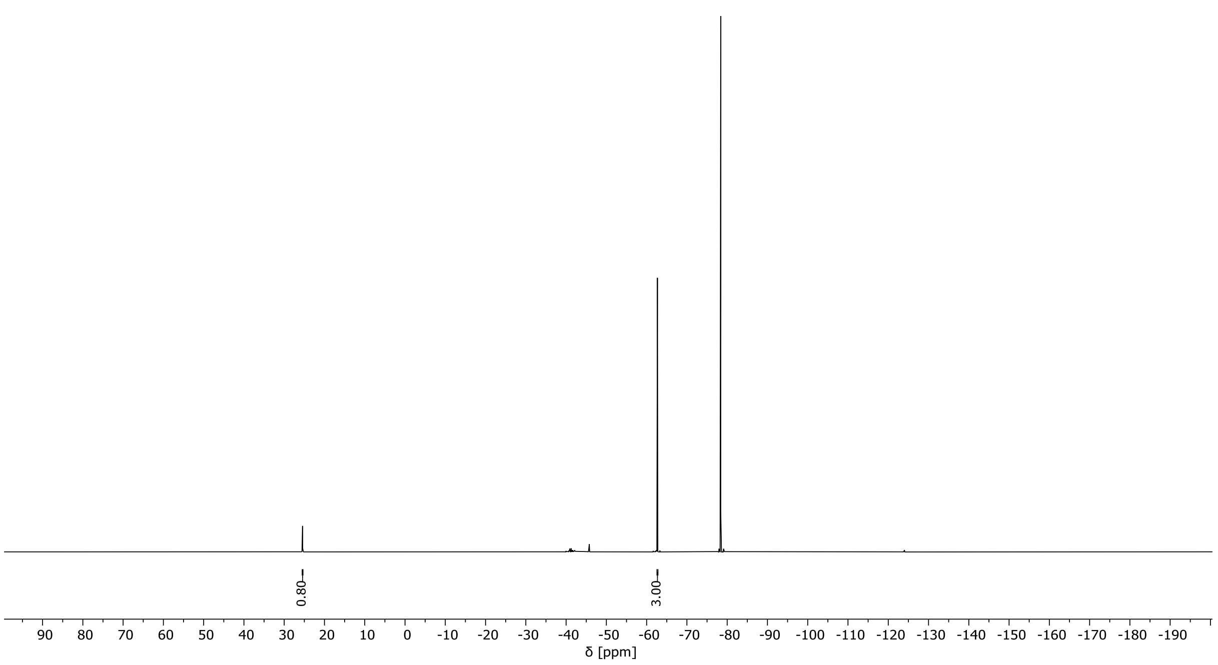

Crude ^{19}F NMR (282 MHz in $CDCl_3$), 298 K

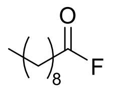

2j

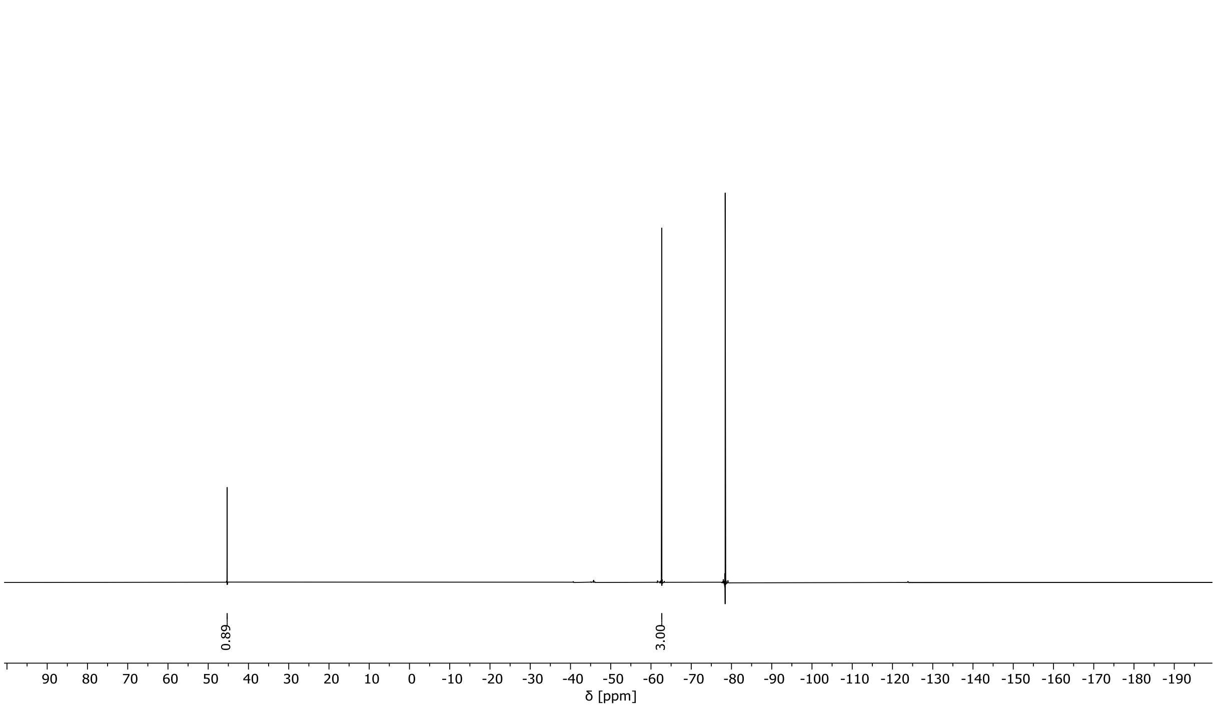

Crude ¹⁹F NMR (282 MHz in $CDCl_3$), 298 K

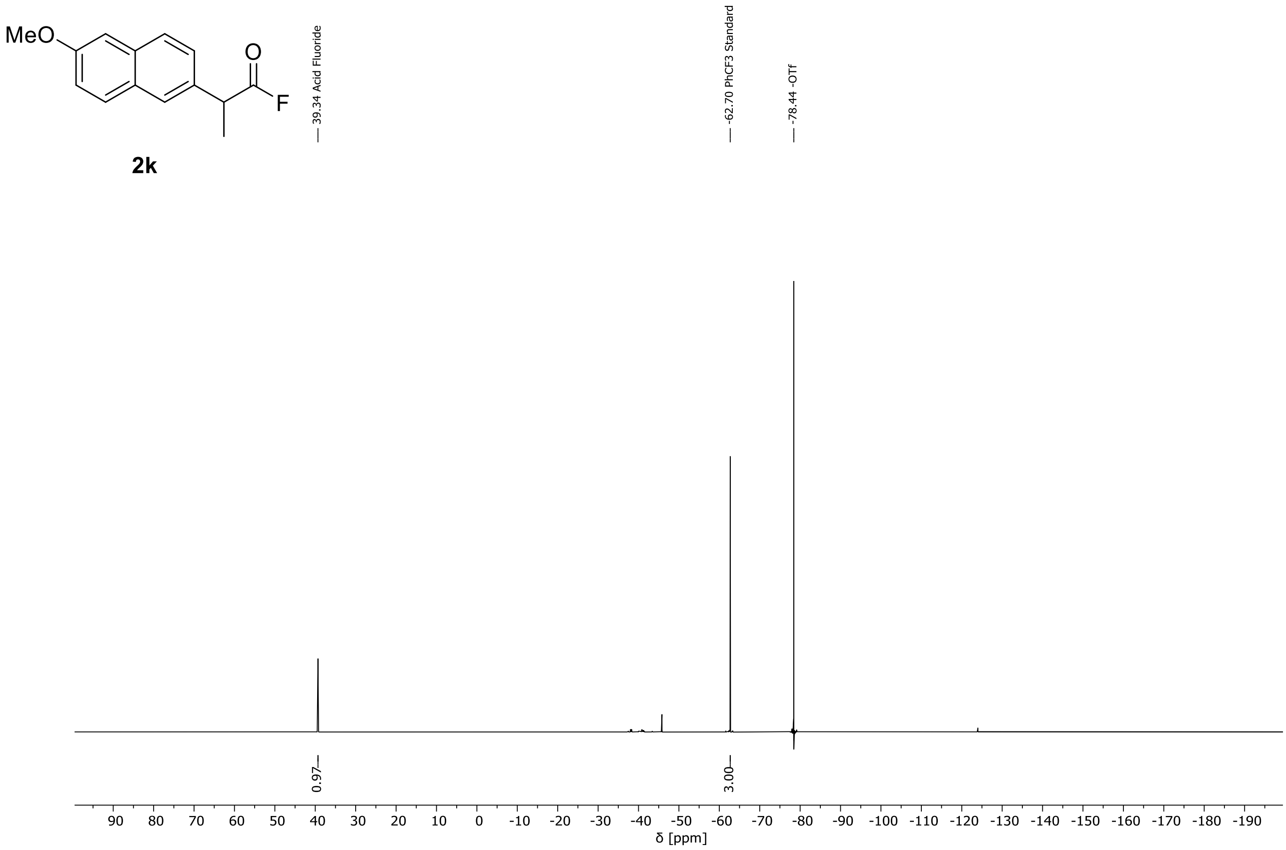

2k

Crude ¹⁹F NMR (282 MHz in $CDCl_3$), 298 K

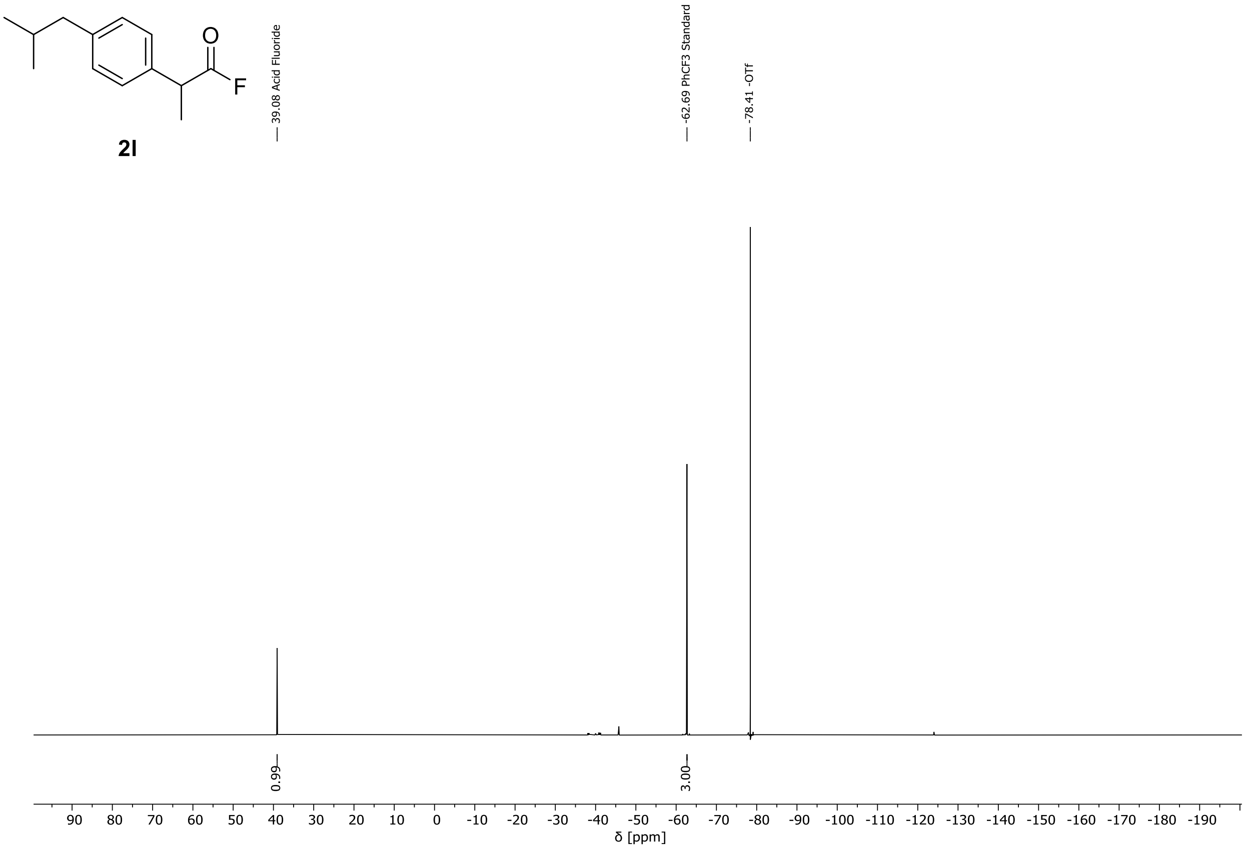

2l

8 NMR Spectra of literature-unknown product 6

^{1}H NMR (400 MHz in $CDCl_3$), 298 K

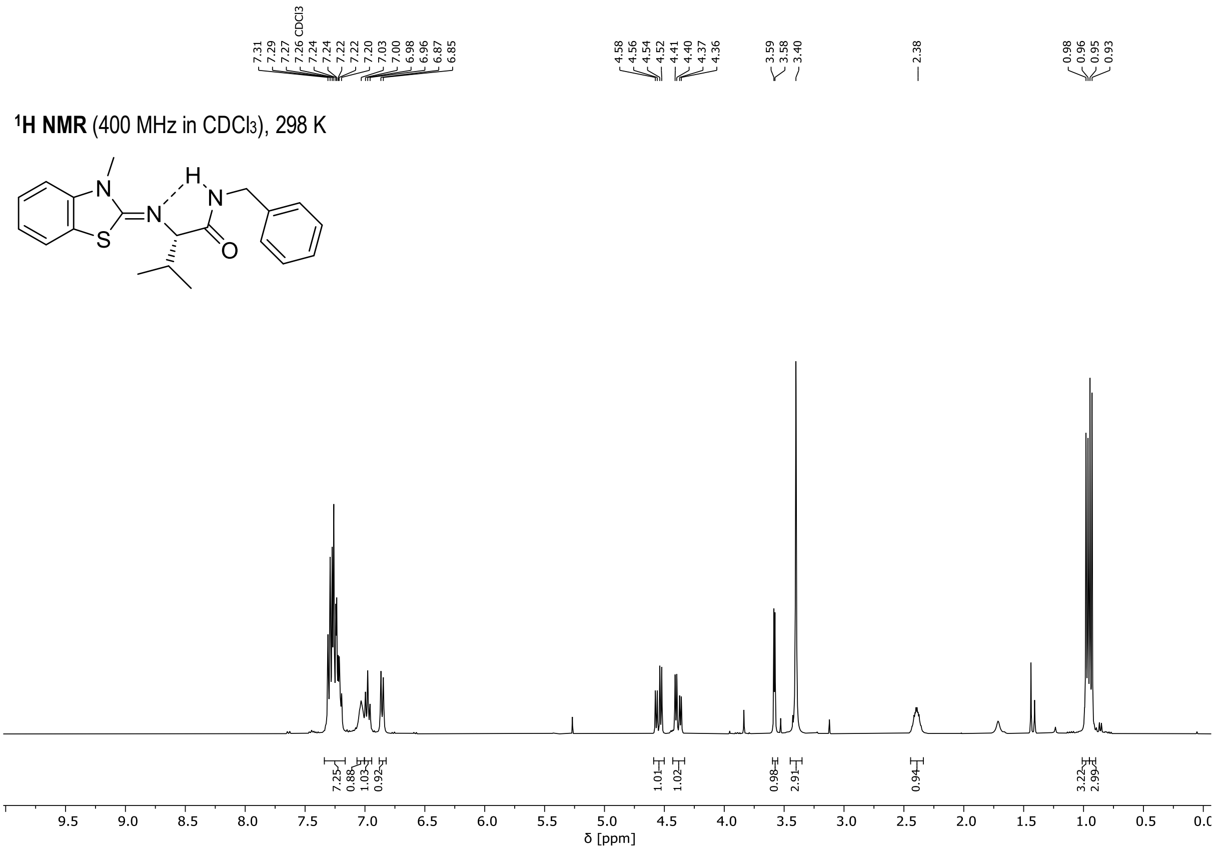

^{13}C NMR (101 MHz in $CDCl_3$), 298 K

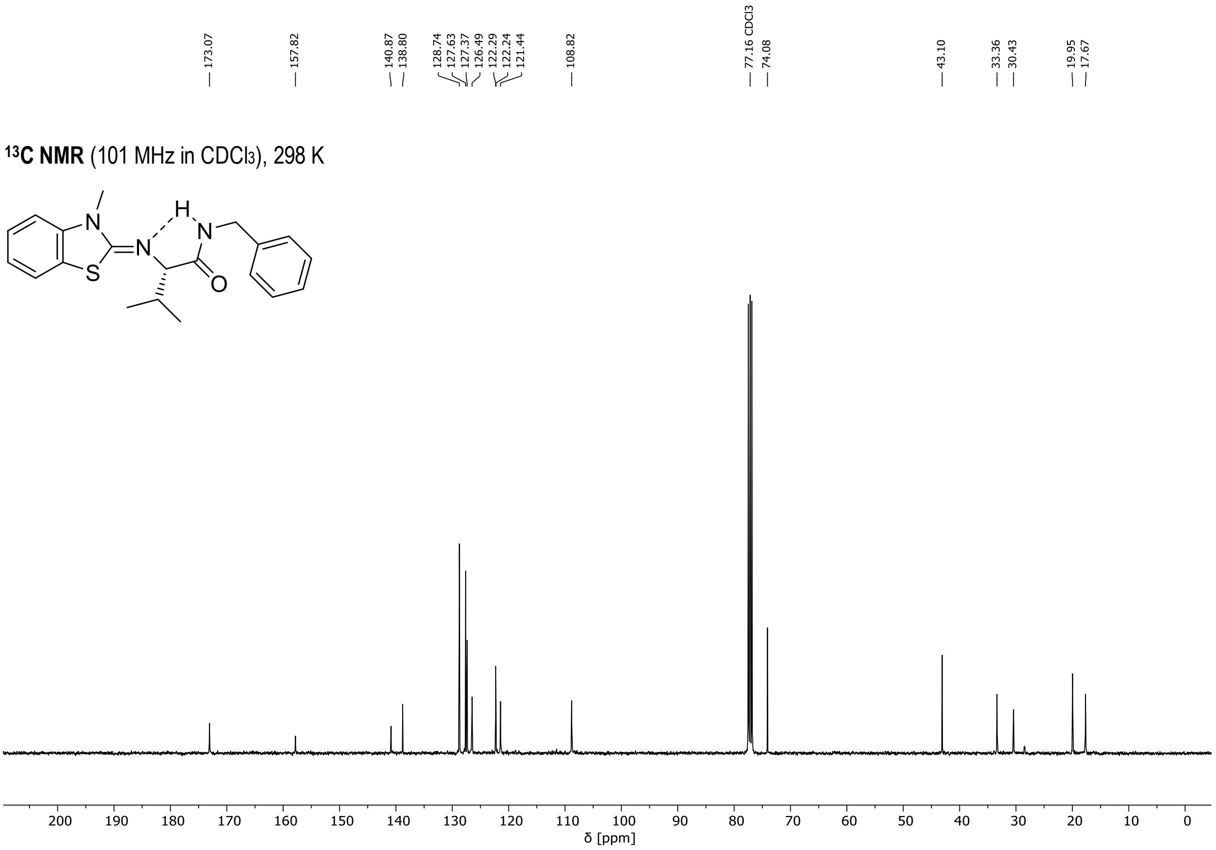

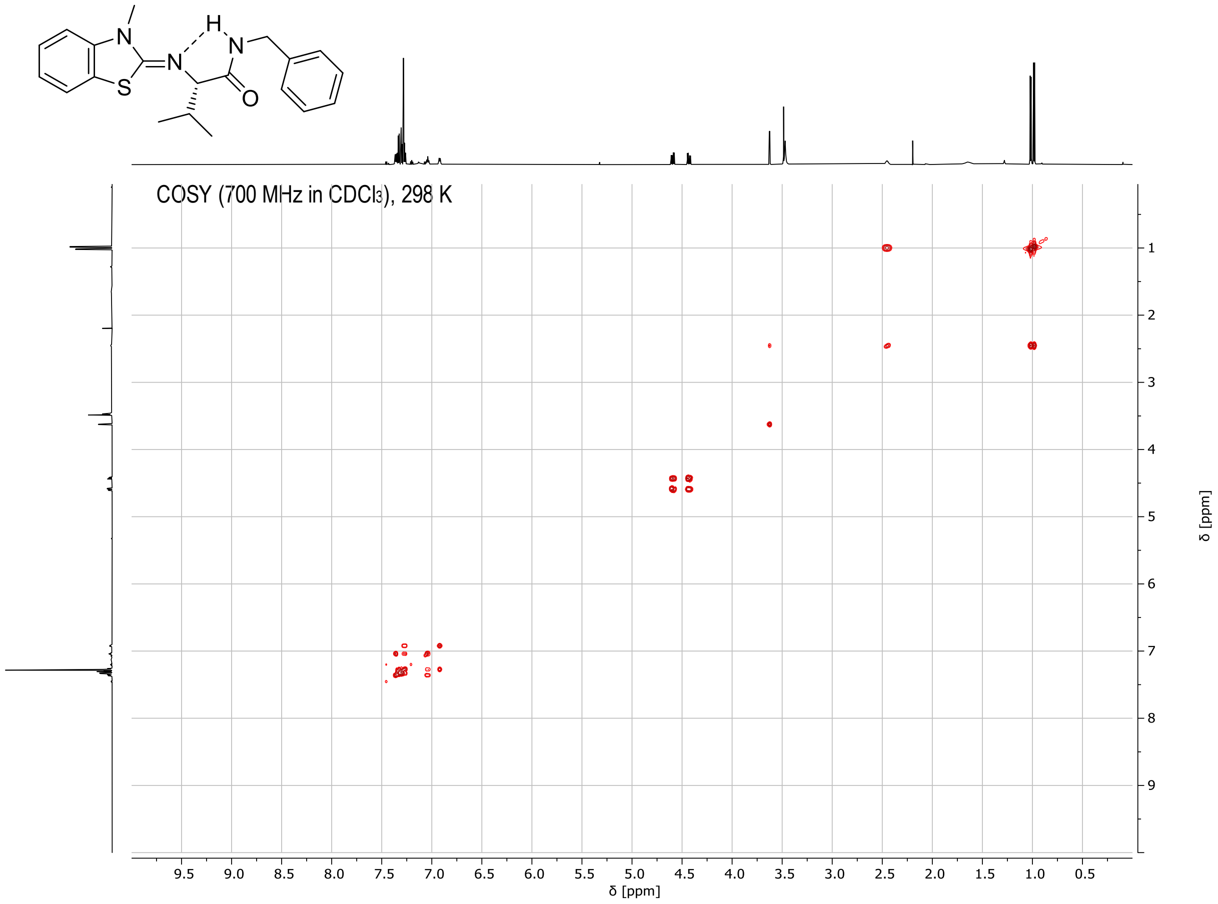
COSY (700 MHz in CDCl3), 298 K
δ [ppm]
δ [ppm]

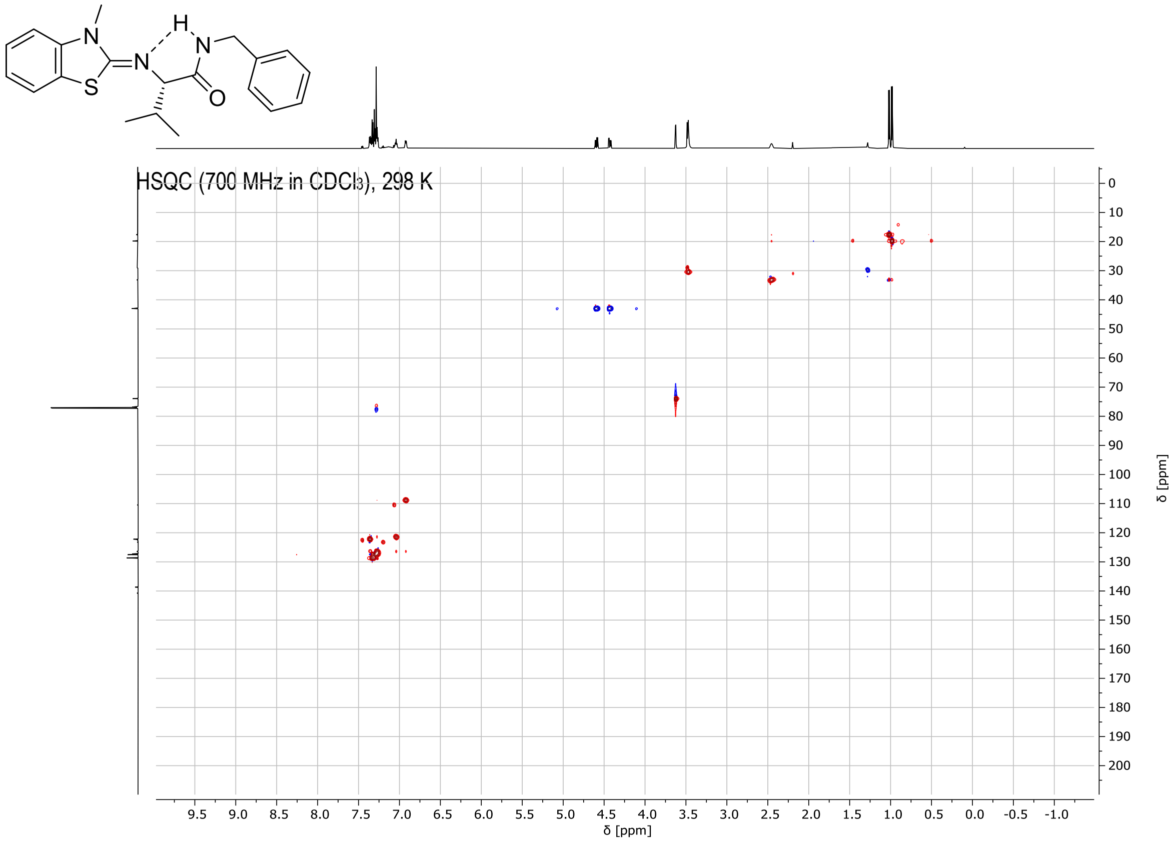
HSQC (700 MHz in CDCl3), 298 K
δ [ppm]
δ [ppm]

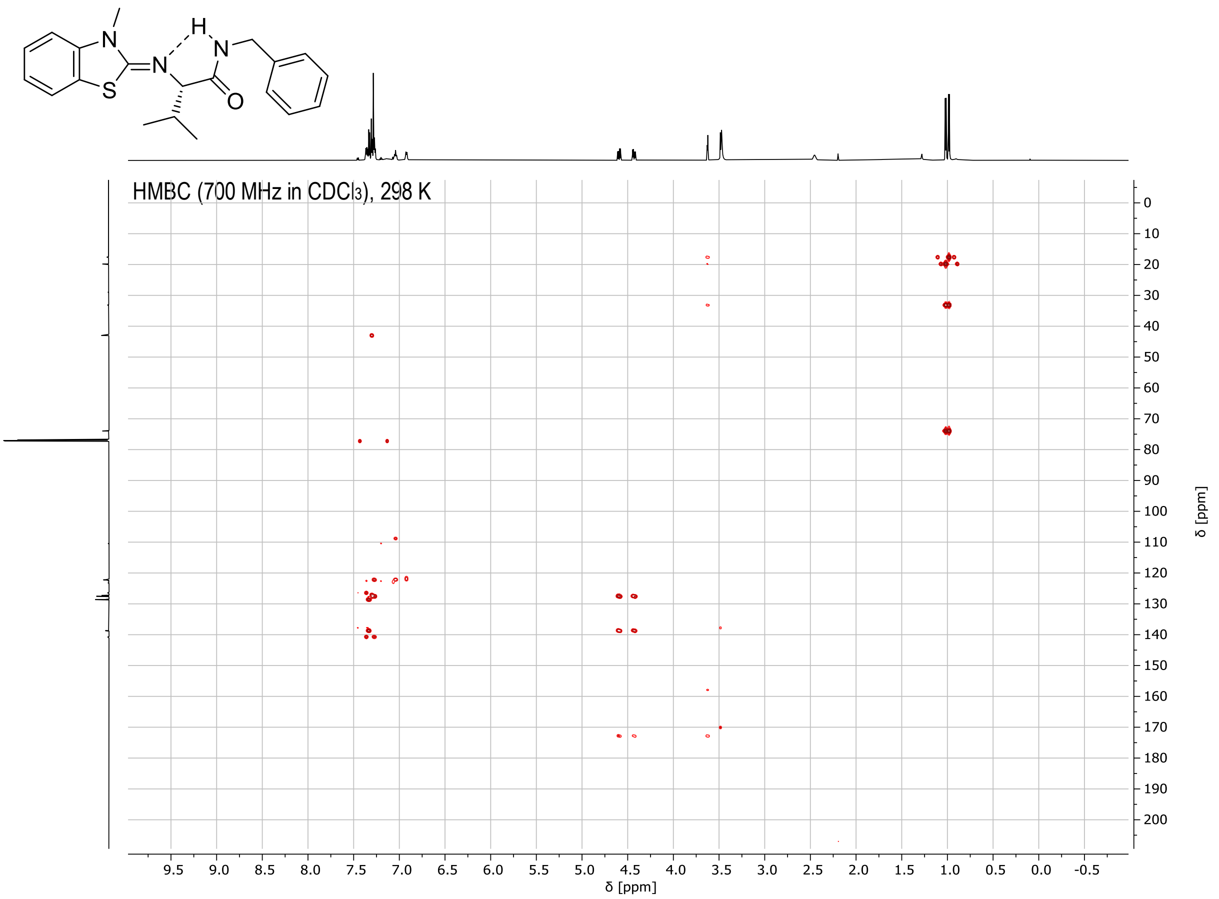

9 References

[1] a) S. Dix, M. Jakob, M. N. Hopkinson, *Chem. Eur. J.* **2019**, *25*, 7635-7639; b) A. Ariamajd, N. J. Gerwien, B. Schwabe, S. Dix, M. N. Hopkinson, *Beilstein J. Org. Chem.* **2021**, *17*, 83-88.

[2] a) M. Baumann, I. R. Baxendale, L. J. Martin, S. V. Ley, *Tetrahedron* **2009**, *65*, 6611-6625; b) M. Arisawa, T. Yamada, M. Yamaguchi, *Tetrahedron Lett.* **2010**, *51*, 6090-6092; c) T. Scattolin, K. Deckers, F. Schoenebeck, *Org. Lett.* **2017**, *19*, 5740-5743; d) M. Meanwell, J. Lehmann, M. Eichenberger, R. E. Martin, R. Britton, *Chem. Commun.* **2018**, *54*, 9985-9988; e) Z. Wang, X. Wang, Y. Nishihara, *Chem. Commun.* **2018**, *54*, 13969-13972; f) S. B. Munoz, H. Dang, X. Ispizua-Rodriguez, T. Mathew, G. K. S. Prakash, *Org. Lett.* **2019**, *21*, 1659-1663.

[3] a) V. C. Agwada, *J. Chem. Eng. Data* **1982**, *27*, 479-481; b) P. V. Ramachandran, H. J. Hamann, *Org. Lett.* **2021**, *23*, 2938-2942.

[4] G. Li, M. Li, Z. Xia, Z. Tan, W. Deng, C. Fang, *J. Org. Chem.* **2022**, *87*, 8884-8891.

[5] N. D. Kokare, R. R. Nagawade, V. P. Rane, D. B. Shinde, *Synthesis*, **2007**, *2007*, 766-772.

[6] J. Wannberg, M. Larhed, *J. Org. Chem.* **2003**, *68*, 5750-5753.

[7] X. Cui, Y. Zhang, F. Shi, Y. Deng, *Chem. Eur. J.* **2011**, *17*, 1382-1382.

[8] J. P. Hibbard, J. G. Yam, E. B. Alsalek, A. Bahamonde, *J. Org. Chem.* **2022**, *87*, 12036-12040.

[9] H. Morimoto, R. Fujiwara, Y. Shimizu, K. Morisaki, T. Ohshima, *Org. Lett.* **2014**, *16*, 2018-2021.

[10] Y. Kawagoe, K. Moriyama, H. Togo, *Tetrahedron*, **2013**, *69*, 3971-3977.

[11] H. Cheng, M.-Q. Xiong, C.-X. Cheng, H.-J. Wang, Q. Lu, H.-F. Liu, F.-B. Yao, C. Chen, F. Verpoort, *Chem. Asian J.* **2018**, *13*, 440-448.

[12] C. R. Opie, H. Noda, M. Shibasaki, N. Kumagai, *Chem. Eur J.* **2019**, *25*, 4648-4653.

[13] a) K. Wang, Y. Lu, K. Ishihara, *Chem. Commun.* **2018**, *54*, 5410-5413; b) F. Almetwali, J. Rouden, J. Blanchet, *Eur. J. Org. Chem.* **2023**, *26*, e202300720.

[14] K. C. Nadimpally, K. Thalluri, N. B. Palakurthy, A. Saha, B. Mandal, *Tetrahedron Lett.* **2011**, *52*, 2579-2582.

[15] D. Riemer, P. Hirapara, S. Das, *ChemSusChem* **2016**, *9*, 1916-1920.

[16] F. Liang, Q. Liu, J. Tan, C. Piao, *Synthesis*, **2008**, *2008*, 3579-3584.
